# Multifunctional MXene-Based Nanomaterials in Optoelectronics: From Interfacial Engineering to Device

**DOI:** 10.3390/mi17080970

**Published:** 2026-08-17

**Authors:** Seongeun Byeon, Seonhu Jung, Junseo Lee, Seongheon Jeon, Seokyeong Lee

**Affiliations:** Department of Electronic Engineering, Kyonggi University, Gwanggyosan-ro, Yeongtong-gu, Suwon-si 16227, Gyeonggi-do, Republic of Korea; byseeu1122@gmail.com (S.B.); seonhu.jj@gmail.com (S.J.); wnstj5928@gmail.com (J.L.); wjstmsgjs@gmail.com (S.J.)

**Keywords:** MXenes, optoelectronics, interfacial engineering, surface terminations, energy-level alignment

## Abstract

Two-dimensional transition-metal carbides and nitrides (MXenes) are increasingly adopted in advanced electronic devices, where their metallic conductivity, optical tunability, and chemically addressable surfaces support next-generation multifunctional optoelectronics. Their practical performance, however, depends not only on their intrinsic properties but also on the heterogeneous interfaces where charges, photons, and ions interact. Unlike earlier reviews organized around synthesis routes or separate device categories, this review takes interfacial chemistry as a single organizing principle and follows it from surface terminations through to integrated systems. The structural and surface-chemical characteristics of MXenes are described first, showing how dynamic terminations and interfacial dipoles regulate work functions and energy-level alignment. We then discuss molecular functionalization, defect passivation, and heterojunction formation as strategies for reducing Schottky barriers and improving charge-transfer kinetics. Optoelectronic platforms built on these engineered interfaces, including high-efficiency photovoltaics, broadband photodetectors, and stretchable wearable systems, are subsequently detailed, together with emerging architectures that merge self-powered sensing with neuromorphic visual functions, a scope seldom treated alongside conventional devices in previous surveys. By connecting surface chemistry with device integration, this review outlines a materials-to-systems pathway toward more reliable and scalable MXene-based optoelectronic technologies.

## 1. Introduction

Optoelectronic technology has advanced quickly in recent years, and with it the way light, electricity, and information are generated, transported, detected, and interpreted. Transparent electrodes [1,2,3,4,5,6], photovoltaics [7,8,9,10,11,12,13,14], photodetectors [15,16,17,18,19,20,21,22,23,24,25,26,27,28,29], light-emitting devices [3,4,30,31,32], wearable sensors [33,34,35,36], and neuromorphic visual systems [37,38,39] are rarely developed in isolation anymore. They are instead combined into platforms that convert energy, transduce signals, carry optical communication [40], and interact directly with users. Such integration places heavy demands on the constituent materials, which must deliver high electrical conductivity, optical tunability [28,41], mechanical compliance [34,35], solution processability [41,42], and chemically programmable interfaces at the same time. Conventional transparent conductors and interfacial layers, including indium tin oxide, noble metals, metal oxides, and carbon-based nanomaterials, have contributed substantially to the development of modern optoelectronics [27,30]. Nevertheless, their intrinsic limitations, such as mechanical brittleness [32], limited work function tunability [14,30], interfacial incompatibility, high processing cost, or insufficient chemical adaptability, continue to restrict the realization of flexible, scalable, and multifunctional optoelectronic systems [31,39].

Two-dimensional transition-metal carbides, nitrides, and carbonitrides, collectively termed MXenes, have emerged as a distinctive class of materials that may bridge this gap [40,43,44]. First synthesized in 2011 from the Ti_3_AlC_2_ MAX phase, MXenes follow the general formula M_n+1_X_n_T_x_, where M is an early transition metal, X is carbon or nitrogen, and T_x_ represents surface termination groups [45]. Unlike semiconducting two-dimensional materials, MXenes possess energy bands that cross the Fermi level [46,47], enabling metal-like charge transport even in optically semi-transparent ultrathin films [43,48,49,50]. MXenes also disperse readily in water or polar solvents as colloids, which opens the way to low-temperature spray [51], blade, and inkjet deposition [52], while their laminar stacking allows the interlayer spacing and the dielectric response to be adjusted by intercalation [53,54]. These characteristics position MXenes at an intersection of conductivity, optical activity, and chemical addressability that the optoelectronics field had previously been unable to access within a single material system [40,49,55,56].

These claims are best assessed against the two-dimensional materials that preceded MXenes. Graphene owes its conduction to *π* bonding within the sp^2^ lattice and reaches a resistivity near 10^−8^ Ω m, but only in ideal single-layer form rather than in the solution-processed films on which scalable fabrication depends. Graphene oxide falls well short of even that practical level, since the rise in sp^3^ character removes the conducting *π* network and drives the resistivity to roughly 10^8^ Ω m, so a thermal or chemical reduction step is required before the material conducts at all. Ti_3_C_2_T_x_ needs no such recovery step and reaches 15,100 S/cm in solution-cast film, the highest value reported for a solution-processed two-dimensional solid [57,58,59]. The same contrast holds in transparent-electrode geometry, where aligned Ti_3_C_2_T_x_ flakes transmitting above 90% of visible light give a sheet resistance near 1032 Ω/sq, about one-third of the graphene value at comparable transparency. More important here, these figures are not fixed by the parent composition. Termination chemistry alone carries the work function from 1.6 eV for hydroxyl-terminated Sc_2_C to 4.60 eV for Ti_3_C_2_ and 5.1 eV for an oxygen-rich surface, a span graphene cannot match while pinned near 4.5 eV [60,61,62].

The optoelectronic relevance of MXenes, however, is not limited to their metallic conduction [63,64]. MXene films exhibit composition-dependent extinction [51,55,65], transmittance, and plasmonic resonances spanning the ultraviolet to the mid-infrared [44,56,66,67,68], all of which shift with flake thickness [69,70], carrier concentration, and surface termination without requiring changes in chemical composition [47,65,71,72,73]. More consequentially for device integration, surface terminations such as –O, –OH, –F, and –Cl alter both the local dielectric environment and the Fermi level simultaneously, coupling the photonic and electronic responses of MXene into a single tunable system [47,54,65,74,75,76,77,78,79,80,81,82,83,84]. This built-in coupling between photons and electrons sets MXenes apart from ordinary conductive additives. It also means they are better regarded as optically active metallic two-dimensional materials, with optoelectronic functions that can be engineered on purpose rather than simply taken advantage of [68,85].

The chemistry of the heterogeneous interface, however, carries the greatest consequence for the integration of MXenes into optoelectronic devices [86]. Surface terminations should not be regarded as passive residues of the synthesis [87,88]. Theoretical and experimental studies agree that the work function of Ti_3_C_2_T_x_ can be shifted reproducibly over a wide range by controlling the termination composition [80,84,87,89,90,91,92,93], by noncovalent molecular doping [94,95,96], or by covalent functionalization with electron-withdrawing or electron-donating organic groups [97,98,99,100,101]. Such adjustments carry immediate consequences for device physics. A mismatch in energy-level alignment at a semiconductor heterojunction may turn an Ohmic contact into a rectifying Schottky junction or introduce Fermi-level pinning that quenches the photovoltage, whereas accurate alignment permits selective carrier extraction and suppresses recombination [87,101,102,103,104,105,106]. The influence of MXene surfaces is not confined to energy-level engineering. Lewis-acidic Ti centers and oxygen-containing terminations coordinate with unpassivated surface metal ions [13,100,107], fill vacancies in oxide charge transport layers [108,109], and chelate dangling bonds, thereby suppressing nonradiative recombination and retarding ionic migration at the same time. This dual character, an electronically adaptive contact that also acts as a chemically passivating interfacial layer, separates MXene surface engineering from conventional conductive interlayers and has brought interfacial design to the center of high-performance MXene optoelectronics [110].

These interfacial principles have been examined across a broad range of device platforms. MXene-modified charge transport layers in photovoltaics [111] suppress recombination and stabilize the interfaces, with gains in both efficiency and operational lifetime [7,8,9,10,11,12,13,14]. Photodetectors exploit the work function contrast at MXene–semiconductor Schottky junctions, where the resulting built-in electric fields support self-powered broadband operation from the ultraviolet to the near-infrared [15,16,17,18,19,20,21,22,23,24,25,27,29,32,35]. Light-emitting and flexible display systems benefit from MXene electrodes of tunable work function, which assist carrier injection while their mechanically compliant film-forming behavior sustains operation under repeated deformation [3,4,30,31,33,49]. Neuromorphic architectures make use of MXene-derived dielectric layers that provide photocarrier trapping and multilevel conductance modulation, emulating biological synaptic plasticity [38,39]. Considerable obstacles nevertheless persist. Ambient oxidation degrades both the conductivity and the reproducibility of the surface dipole [33,39], fluoride-based synthesis yields stochastic termination distributions that complicate interfacial control [14,38], and the formulation of scalable inks with suppressed interlayer restacking calls for further process engineering [26,34,42].

**Figure 1:**
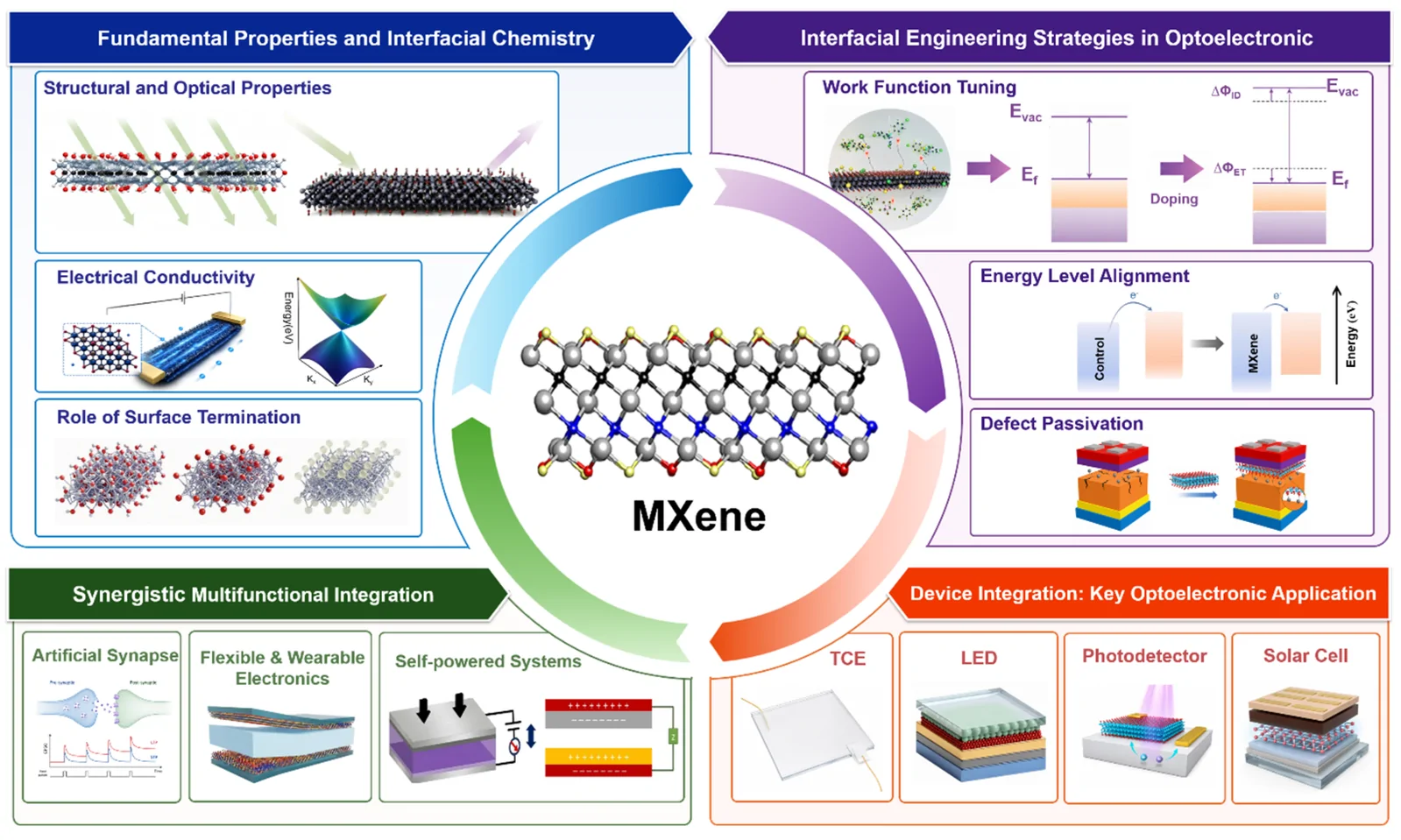
Schematic illustration of interfacial engineering and optoelectronic applications of multifunctional MXenes.

The present review surveys recent progress in multifunctional MXene-based materials, taking as its unifying theme the transition from fundamental interfacial engineering to robust device integration, as outlined schematically in Figure 1. Structural and surface-chemical foundations are treated first, followed by work function tuning [30,31,32,93], energy-level alignment [13,96], Schottky barrier modulation [27,29,41,101,104,112], and defect passivation strategies [13,38,104,108,109]. Representative device platforms are then considered, covering photovoltaics [7,8,9,10,11,12,14], photodetectors [15,16,17,18,19,20,21,22,23,24,25,29,34], light-emitting systems [3,4,30,31], wearable optoelectronics [33,36], and neuromorphic visual interfaces [28,37,39,41]. A concluding perspective addresses fluorine-free synthesis, oxidation-resistant design, and scalable processing toward reliable MXene-based optoelectronic technologies [105,113].

## 2. Fundamental Properties and Interfacial Chemistry

### 2.1. Optical and Plasmonic Properties

The optical response of MXenes arises from the interplay between the free carriers of a metallic transition-metal carbide or nitride lattice and the surface terminations that cover each atomically thin sheet, both of which can be tailored through composition and processing [114,115,116]. This tunability endows MXenes with an optical behavior that spans transparent conduction [48,50,115], broadband absorption [71,117], and wavelength-selective reflection [46,117] across the ultraviolet, visible, and infrared, making them attractive for optoelectronic and photothermal devices while also complicating the interpretation of their characteristic spectral features [40,118,119]. Establishing how these features depend on composition, carrier density, and surface chemistry is therefore a prerequisite for their rational design.

**Figure 2:**
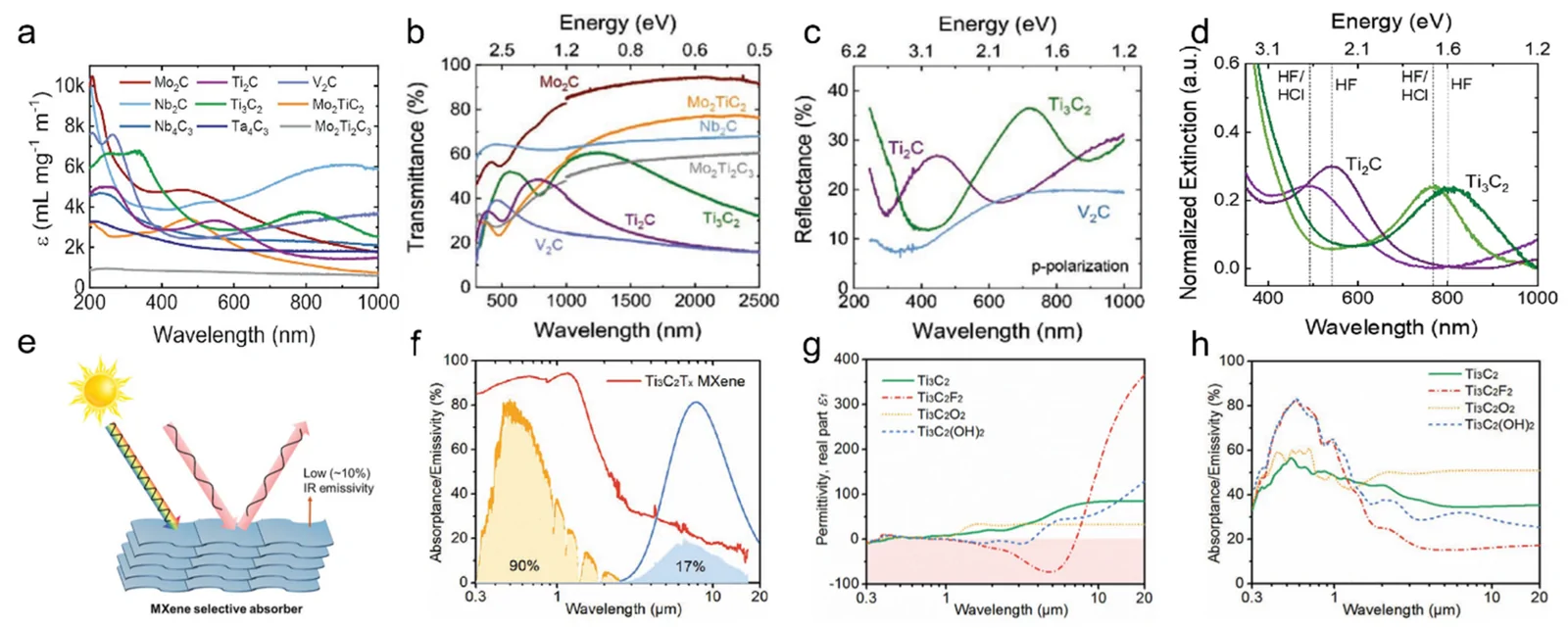
(**a**) UV-vis-NIR optical extinction properties of 2D transition metal carbides measured for colloidal solutions. (**b**) UV-vis-NIR transmittance spectra for MXene thin films. (**c**) Reflectance spectra of Ti_3_C_2_, Ti_2_C, and V_2_C films measured at a 75° incident angle using *p*-polarized light. (**d**) Normalized extinction spectra of Ti_3_C_2_ and Ti_2_C colloidal solutions synthesized *via* HF/TMAOH or HF/HCl/LiCl. Reproduced with permission from Ref. [51]. Copyright (2020), Wiley-VCH GmbH. (**e**) Schematic illustration of the high solar absorptance, high mid-infrared reflectance, and low infrared emissivity of MXene. (**f**) Absorptance/emissivity spectrum of Ti_3_C_2_T_x_ MXene (red) with solar (yellow) and thermal radiation (blue) spectra. (**g**) Real (*ε*_1_) part of the in-plane permittivity of Ti_3_C_2_T_x_ with different surface terminations. (**h**) Absorptance/emissivity spectra of Ti_3_C_2_T_x_ with different terminations. Reproduced with permission from Ref. [117]. Copyright (2021), Wiley-VCH GmbH.

Gogotsi and co-workers surveyed nine carbide MXenes and showed that their optical response is governed primarily by composition (Figure 2a–d). The gravimetric extinction spectra display a strong, compositionally insensitive absorption in the UV together with a characteristic visible-to-NIR peak whose position is set by the atomic structure. Reducing the layer number from Ti_3_C_2_ to Ti_2_C, or substituting Ti with Mo, systematically pushes this peak toward higher energy (Figure 2a). The thin-film transmittance divides the MXenes into two families (Figure 2b). In one group, the NIR transmittance increases with wavelength, whereas in the other, it decreases. This latter group, comprising the highly conductive V_2_C, Ti_2_C, and Ti_3_C_2_, shows a negative slope indicative of metallic reflection that emerges near the wavelength at which the real part of the dielectric function turns negative, as expected for a Drude-like free-carrier plasma. This interpretation is supported by *p*-polarized reflectance (Figure 2c), where the onset of strong reflection coincides with the decline in transmittance.

The reflectance maximum of Ti_3_C_2_ moreover aligns closely with the plasmon energy-loss peak obtained from STEM-EELS, pointing to a common plasmonic origin for the reflectance and extinction features. A key prediction of this plasmonic picture is that the peak should shift to higher energy as the free-carrier density rises. In accordance with this, replacing the conventional HF etchant with a mixed-acid (HF/HCl) route and LiCl intercalation blue-shifts the extinction peaks of both Ti_2_C and Ti_3_C_2_ (Figure 2d), consistent with a carrier-concentration–dependent plasmon resonance [51].

Beyond broadband optical absorption, MXenes exhibit pronounced spectral selectivity that enables efficient solar–thermal conversion with suppressed infrared heat loss. Li et al. demonstrated that Ti_3_C_2_T_x_ behaves as a visible-black but infrared-white selective absorber, strongly absorbing incident solar radiation while reflecting mid-infrared thermal radiation (Figure 2e). The freestanding Ti_3_C_2_T_x_ film exhibited a solar absorptance of approximately 90% and a thermally weighted infrared emissivity of only 17% at 100 °C (Figure 2f), with values as low as approximately 10% achieved for surfaces with more favorable nanosheet alignment. This combination reduces radiative heat loss and improves solar–thermal energy retention without requiring a lithographically patterned metasurface.

First-principles calculations further revealed that this spectral selectivity is strongly governed by surface termination (Figure 2g,h). The complex permittivity of uniformly terminated Ti_3_C_2_T_x_ models was obtained from density functional theory with a hybrid functional, and the normal-incidence reflectance was evaluated through the Fresnel relation, with the absorptance taken as unity minus the reflectance for optically thick films. Because the electric field lies in the basal plane, the in-plane dielectric response dominates reflection from the layered film. The real permittivity of Ti_3_C_2_F_2_ and Ti_3_C_2_(OH)_2_ becomes negative in the near-infrared region and increases substantially in magnitude at longer wavelengths, indicating a strong metallic response that enhances mid-infrared reflection. These fluorine- and hydroxyl-terminated MXenes therefore retain high absorption across the solar spectrum while exhibiting substantially lower infrared emissivity than pristine Ti_3_C_2_ and Ti_3_C_2_O_2_. Such models nevertheless assume a single termination species on an ideal periodic surface, whereas etched films carry disordered mixtures of −O, −OH, and −F together with vacancies and intercalated water, which explains why the predicted spectra reproduce experimental trends without matching them quantitatively. Quantitative prediction will require configurational averaging over mixed terminations, along with validation against measured optical constants [117].

Additionally, the physical origin of the near-infrared extinction feature in Ti_3_C_2_T_x_ remains under debate. Spectroscopic ellipsometry, combined with density functional theory in which the interband optical conductivity is constructed from maximally localized Wannier functions and the intraband part is added through a Drude term, attributes the broad feature near 1.5 eV to an interband transition rather than to a localized plasmon resonance. The transition connects nearly flat bands close to the Γ point of oxygen-terminated Ti_3_C_2_O_2_, and the only well-defined surface plasmon polariton is predicted near 0.5 eV, outside the optical window. Supporting evidence comes from the near disappearance of the feature in chlorine-terminated Ti_3_C_2_Cl_2_ and from the positive real permittivity measured at 1.5 eV, which cannot sustain a plasmonic mode. Because such models assume a single termination species and an empirically fixed Hubbard correction for the Ti 3d states, they describe limiting cases rather than films with mixed terminations. The optical spectra of MXenes therefore arise from an interplay between free-carrier and termination-dependent interband contributions rather than from a single universal plasmonic mechanism [65].

Beyond surface termination, the optical response of MXenes can also be regulated through the X-site composition of the lattice. First-principles calculations of Ti_3_CN, with optical spectra evaluated within the Tamm–Dancoff approximation to time-dependent density functional theory, show that partial substitution of carbon by nitrogen preserves metallic band dispersion while leaving a comparatively flat conduction band just above the Fermi level. Parallel band absorption between these features sustains strong broadband absorption extending into the infrared, and the response is further differentiated by O- and F-based terminations. Nonadiabatic molecular dynamics on the same structures links this band arrangement to carrier dynamics, since fluorine- and mixed-terminated models release most of the excess electron energy within roughly 200 fs whereas the O-terminated surface relaxes far more slowly. X-site substitution and termination chemistry thus provide complementary routes for tailoring the electronic and optical characteristics of MXenes [118]. The optical and plasmonic properties of the representative MXenes discussed above are summarized in Table 1, which compiles their composition, characteristic extinction or reflectance features, and the resulting optical functionality across the ultraviolet, visible, and infrared regions.

### 2.2. Electrical Transport

The same metallic free-carrier system that governs the optical response of MXenes also makes them among the most conductive of all solution-processable two-dimensional materials, underlying their use as electrodes and charge transport layers [50]. This conductivity, however, is not a fixed material constant but is shaped by the transition-metal chemistry and atomic-layer structure [79,120,121], by the surface terminations [80,83,90,122] and confined interflake water [65,69,123,124], and by the degree of oxidation [69,79,125], so that thermal history and processing can either enhance or degrade transport [123,126,127]. Because the optical and electrical responses share a common free-carrier origin [44,46,122,128,129], spectroscopic probes provide a sensitive, contactless route to tracking how these properties evolve during processing [64,125].

**Figure 3:**
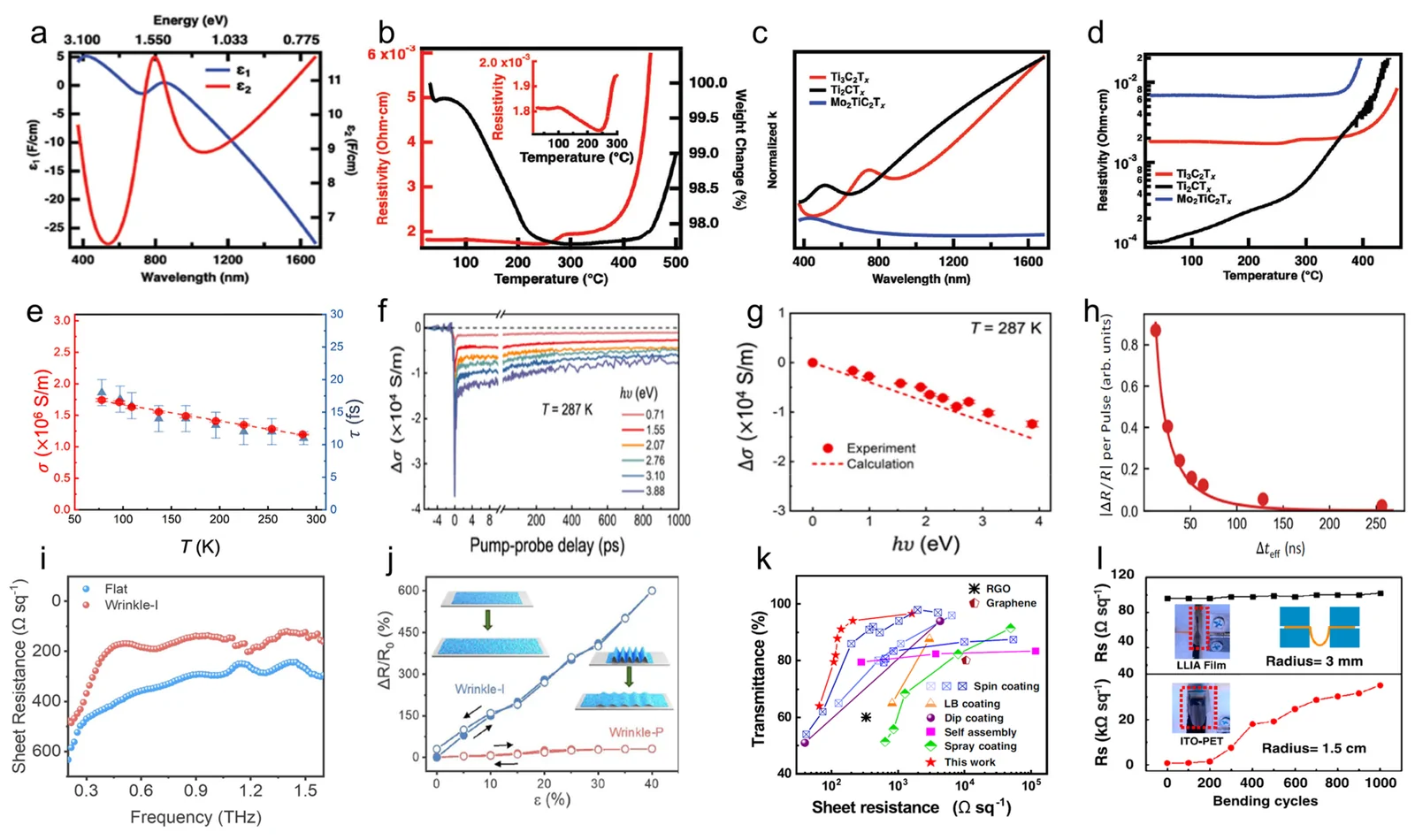
(**a**) Real (*ε*_1_) and imaginary (*ε*_2_) components of the dielectric constant of Ti_3_C_2_T_x_ (14 nm film) as a function of wavelength. (**b**) Optical resistivity and TGA weight change in a Ti_3_C_2_T_x_ film (19 nm film) as a function of temperature. (**c**) Normalized extinction coefficient (**k**) spectra of Ti_3_C_2_T_x_ (19 nm film), Ti_2_CT_x_ (3 nm film), and Mo_2_TiC_2_T_x_ (9 nm film). (**d**) Temperature-dependent optical resistivity of Ti_3_C_2_T_x_, Ti_2_CT_x_, and Mo_2_TiC_2_T_x_. Reproduced with permission from Ref. [69]. Copyright (2023), Wiley-VCH GmbH. (**e**) Conductivity (*σ*) and scattering time (*τ*) of Ti_3_C_2_T_x_ films as a function of temperature. (**f**) Time-dependent photoconductivity changes in Ti_3_C_2_T_x_ for various photon energies (*hv*) at 287 K. (**g**) Average photoconductivity extracted from the 3–10 ps delay time range in (**f**) as a function of photon energy. (**h**) Accumulated transient reflectivity (Δ*R/R*) as a function of effective delay time (Δ*t*_eff_). Reproduced with permission from Ref. [130]. Copyright (2026), Springer Nature. (**i**) Terahertz sheet resistance of Flat (uniform film on quartz) and Wrinkle-I (isotropic wrinkles on PDMS) Ti_3_C_2_T_x_ films across 0.2–1.6 THz. (**j**) Resistance changes in the Wrinkle-I and Wrinkle-P films during the loading and unloading processes. Reproduced with permission from Ref. [49]. Copyright (2024), Springer Nature. (**k**) Transmittance at 550 nm versus sheet resistance for Ti_3_C_2_T_x_ thin films fabricated by different methods. (**l**) Sheet resistance stability of LLIA Ti_3_C_2_T_x_ and commercial ITO-PET as a function of bending cycles. Reproduced with permission from Ref. [43]. Copyright (2021), Springer Nature.

Fakhraai et al. [69] investigated the thermal evolution of the optical and electrical properties of MXene thin films using in situ spectroscopic ellipsometry under ambient heating conditions (Figure 3a–d). The measured spectra were reconstructed with a multilayer optical model combining two harmonic oscillators for the interband extinction with a Drude oscillator for the free-carrier response, so that the resistivity is obtained from the same fit that describes the absorption bands. As shown in Figure 3a, the real part of the dielectric function of Ti_3_C_2_T_x_ turns negative near 620 nm, marking the onset of free-carrier oscillations, and Drude fitting of a 14 nm film gives an optical resistivity of 0.0010 ± 0.0001 Ω cm, or 978 ± 10 S/cm. Because the model treats the porous film as an effective medium, this value represents a lower bound on the DC resistivity. Two thermal regimes then emerge from the resistivity and thermogravimetric data (Figure 3b). Heating from about 100 to 245 °C lowers the resistivity through removal of adsorbed and intercalated water, and first-principles calculations attribute the accompanying gain to surface de-functionalization, which raises the density of states at the Fermi level.

Further heating reverses the trend, since the resistivity rises and the extinction falls as the conductive network degrades through de-functionalization and oxidation to TiO_2_. The red-shift of the visible–NIR extinction band during this process argues against a plasmonic assignment, because loss of the water dielectric environment would blue-shift a resonance, and it supports interband transitions between closely spaced surface-derived bands, with the intrinsic plasmon predicted near 10 eV. Comparison of Ti_3_C_2_T_x_, Ti_3_CT_x_, and Mo_2_TiC_2_T_x_ shows that transition-metal composition, atomic-layer thickness, and synthesis-derived intercalants govern both conductivity and stability (Figure 3c,d), with oxidation onsets near 450, 300, and 400 °C, respectively. For Mo_2_TiC_2_T_x_, Drude and Gaussian oscillators fit the data equally well within the accessible spectral window, the latter yielding a 0.06 eV gap close to the value predicted by density functional theory, so the metallic or semiconducting character of this MXene cannot be settled without infrared measurements. Controlled annealing below the oxidation threshold therefore improves conductivity, whereas excessive thermal exposure degrades both optical and electrical performance [69].

Wang et al. [130] investigated photothermal regulation of charge transport in a Ti_3_C_2_T_x_ MXene thin film using terahertz time-domain spectroscopy, optical pump–terahertz probe spectroscopy, and transient reflectance measurements (Figure 3e–h). As shown in Figure 3e, the static terahertz conductivity and carrier scattering time decrease with increasing temperature, whereas the carrier density remains nearly unchanged. Within the Drude framework, σ = Ne^2^τ/m*, this indicates that conductivity is governed mainly by scattering time and carrier mobility, with electron–phonon scattering as the dominant mechanism. Upon optical excitation, photoconductivity rapidly decreases within a few picoseconds and remains suppressed over nanosecond timescales (Figure 3f). The stronger negative photoconductivity at higher pump photon energies (Figure 3g) shows that absorbed optical energy is transferred to the lattice, increasing phonon populations and carrier scattering.

Transient reflectance measurements confirm the unusually slow thermal relaxation of Ti_3_C_2_T_x_, with residual heat persisting beyond 100 ns and a thermal decay constant of approximately 40 ns (Figure 3h). Thus, Ti_3_C_2_T_x_ behaves as an efficient phonon heat reservoir, where rapid carrier-to-lattice energy transfer and slow heat dissipation sustain the elevated lattice temperature and prolonged conductivity suppression. This coupled photothermal–electrical response provides a physical basis for MXene-based photothermal electronics, infrared sensing, thermal management, and optoelectronic devices [130].

Structural engineering of MXene films provides an additional route to improve electrical transport and mechanical reliability without sacrificing optical transparency. Gui et al. demonstrated wrinkled Ti_3_C_2_T_x_ films by forming isotropic wrinkle structures on PDMS substrates and periodic longitudinal wrinkles on pre-stretched PDMS substrates (Figure 3i,j). In the terahertz region, the Wrinkle-I film exhibited a lower sheet resistance than the flat film, indicating that the wrinkled architecture enhances effective charge transport by increasing the carrier density and prolonging the scattering time (Figure 3i). More importantly, the Wrinkle-P film maintained stable electrical pathways under mechanical deformation, showing only a modest resistance increase even at 40% tensile strain (Figure 3j). This behavior suggests that periodic wrinkles can accommodate external strain through structural unfolding, thereby preserving the conductive MXene network and improving the durability of flexible optoelectronic films [49].

For transparent electrode applications, Ling et al. introduced a liquid/liquid interfacial self-assembly method to construct highly uniform Ti_3_C_2_T_x_ films with horizontally aligned nanosheets and minimized interflake overlap (Figure 3k,l). The resulting liquid/liquid interfacial self-assembly (LLIA) Ti_3_C_2_T_x_ electrodes achieved a favorable balance between optical transparency and electrical conductivity, with sheet resistance values of 1623, 210, and 123 Ω/sq at transmittances of 96.9, 94.1, and 87.8%, respectively. These values outperform many MXene films prepared by conventional coating methods as well as graphene-based transparent conductors at comparable transmittance. In addition, the LLIA Ti_3_C_2_T_x_/PET electrode showed excellent bending stability, with only a 6% increase in resistance after 1000 bending cycles at a radius of 3 mm, whereas commercial ITO/PET suffered a three-order-of-magnitude resistance increase under milder bending conditions [43]. These results highlight that controlling MXene film architecture, either through wrinkled structures or interfacial self-assembly, is essential for translating the intrinsic conductivity of MXene nanosheets into mechanically robust and optically transparent device electrodes. Additionally, the major conductivity-dependent variables and their corresponding electrical, optical, thermal, and mechanical characteristics relevant to MXene-based optoelectronic device integration are summarized in Table 2.

### 2.3. Surface Terminations and Energy-Level Control

Owing to their high electrical conductivity, MXenes have attracted considerable attention as electrodes and charge transport layers in a range of optoelectronic devices, including solar cells [9,11], field-effect transistors [131], and light-emitting diodes [31]. In such devices, efficient operation depends not only on the conductivity of the individual layers but also on how their energy levels align at the interfaces [94]. Well-matched energy levels minimize interfacial barriers and maximize charge transport efficiency, which places a premium on precise control of the MXene work function [111]. This work function is governed largely by the surface terminations introduced during etching, since each termination imposes a distinct surface dipole and redistributes interfacial charge, thereby reshaping the electronic structure [73,80,81,84,89,131,132]. Accordingly, considerable effort has been directed toward tailoring MXene surfaces through thermal and chemical treatments, molecular adsorption, and covalent functionalization in order to tune the work function to the values required for a given device architecture [94,97,98,111,131].

Schultz et al. established that the work function of Ti_3_C_2_T_x_ is governed not simply by termination stoichiometry, but by the collective surface dipole generated by coexisting O, OH, and F species. Upon vacuum annealing, removal of adsorbates and OH-containing species increased the work function from approximately 3.9 to 4.8 eV, whereas F desorption above 500 °C reduced it toward 4.1 eV. DFT calculations further showed that a weighted average of uniformly terminated surfaces fails to reproduce experiments, whereas mixed-termination models capture the local dipole distribution and interlayer structural response more realistically [131]. Jing et al. extended this concept from thermally driven termination redistribution to molecularly controlled surface functionalization. Covalent grafting of electron-withdrawing 4-nitrophenyl groups through diazonium chemistry progressively increased the Ti_3_C_2_T_x_ work function from 4.70 to 5.28 eV. The increase originates from modification of the surface electrostatic potential by the nitrophenyl-induced dipole and associated electron withdrawal. Consistently, DFT calculations predicted monotonic work function increases with molecular coverage for both Ti_3_C_2_O_2_ and Ti_3_C_2_(OH)_2_ surfaces. These results establish surface dipole engineering as a direct route for controlling MXene energy-level alignment, from thermally regulated native terminations to chemically programmable molecular interfaces [97].

**Figure 4:**
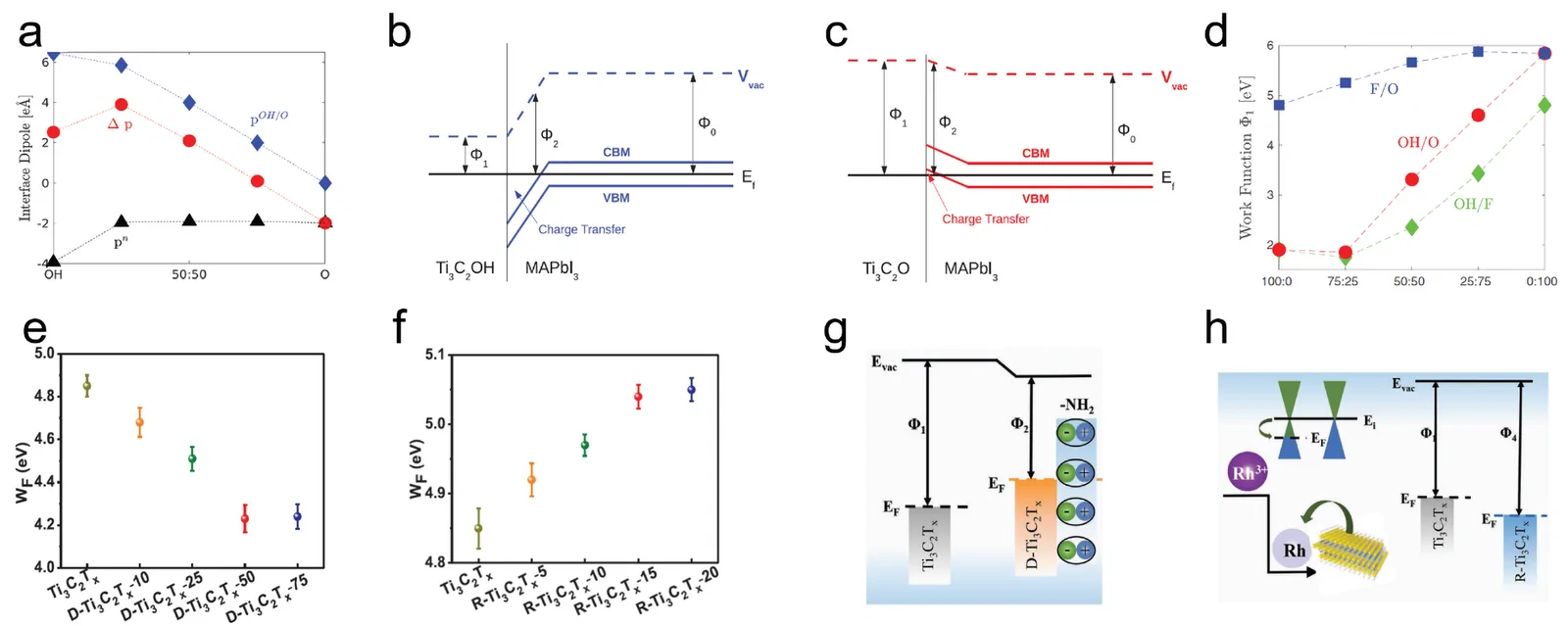
(**a**) Variation in interface dipole Δ*p* (red), adsorbate term *p*^OH/O^ (blue), and electronic rearrangement term *p*^n^ (black) with OH fraction from 100% to 0%. Schematic representation of the band alignment and charge transfer at the (**b**) MAPbI_3_/Ti_3_C_2_OH and (**c**) MAPbI_3_/Ti_3_C_2_O interfaces. (**d**) Work function *Φ* at the perovskite/MXene interface with varying ratios of OH/O, F/O, and OH/F. Reproduced with permission from Ref. [111]. Copyright (2020), Wiley-VCH GmbH. (**e**) Work function of ethanolamine-treated Ti_3_C_2_T_x_ MXene. (**f**) Work function of RHCl_3_-treated Ti_3_C_2_T_x_ MXene. Schematic diagram of work function change mechanisms by surface functionalization: (**g**) D-Ti_3_C_2_T_x_; (**h**) R-Ti_3_C_2_T_x_. Reproduced with permission from Ref. [98]. Copyright (2022), Wiley-VCH GmbH.

Extending these findings to mixed-termination surfaces, Di Vito et al. used DFT calculations to reveal a strongly nonlinear dependence of the Ti_3_C_2_T_x_ work function on the relative fractions of OH, O, and F terminations and further examined its consequences at the MXene/MAPbI3 interface (Figure 4a–d). The work function variation follows the change in the net surface dipole, Δ*Φ* ≈ −Δ*p*, where Δ*p* contains both the intrinsic termination dipole and the opposing contribution from electronic charge rearrangement. As shown in Figure 4a, fully OH-terminated Ti_3_C_2_ exhibits a large adsorbate dipole, but this is substantially screened by redistribution of the surface electron density. Introducing 25% O slightly decreases the termination dipole but markedly weakens the electronic screening, increasing the net dipole from approximately 2.6 to 3.9 eÅ. Consequently, the work function reaches a minimum near the 75:25 OH/O composition rather than at complete OH coverage, demonstrating that mixed terminations generate collective electrostatic responses that cannot be described by linear interpolation.

This nonlinear electrostatic response persists upon contact with MAPbI_3_. For Ti_3_C_2_OH/MAPbI_3_, interfacial charge redistribution produces a dipole that lowers the vacuum level and shifts the perovskite Fermi level toward, and within, the conduction-band region, yielding an interfacial work function of approximately 1.9 eV (Figure 4b). O termination reverses the dipole orientation, drives the Fermi level toward the valence-band region, and increases the work function to approximately 5.8 eV (Figure 4c). Mixed OH/O and OH/F interfaces retain the pronounced nonlinearity, with minima again appearing near 75% OH coverage (Figure 4d). Thus, the interfacial energetics of MXenes are governed by the coupled response of termination dipoles, electronic screening, and interface-induced charge transfer rather than by the independent contributions of individual functional groups. This cooperative electrostatic behavior provides a microscopic basis for tuning carrier-selective energy alignment in MXene/perovskite heterointerfaces [111].

Translating this mechanistic understanding into experimentally accessible bidirectional control, Hou et al. [111] demonstrated a bidirectional work function tuning strategy for Ti_3_C_2_T_x_ MXene through surface treatment with ethanolamine and RhCl_3_ (Figure 4e–h). Kelvin probe measurements showed that ethanolamine treatment progressively decreased the work function of Ti_3_C_2_T_x_ from approximately 4.80 to 4.23 eV as the ethanolamine concentration increased, whereas RhCl_3_ treatment increased the work function to approximately 5.04 eV with increasing RhCl_3_ concentration (Figure 4e,f). This opposite modulation enables Ti_3_C_2_T_x_ to be adapted either as an electron-selective or hole-selective interfacial layer, depending on the energetic requirements of the device.

Mechanistically, the decrease in work function after ethanolamine treatment is attributed to the combined effects of interfacial dipole formation and Fermi-level upshift (Figure 4g). The −NH_2_ groups in ethanolamine interact with the −O, −OH, and −F terminations of Ti_3_C_2_T_x_ through hydrogen bonding, forming dipoles with the negative end directed toward MXene and the positive end directed outward. This dipole lowers the vacuum level, while electron donation from −NH_2_ groups to the MXene surface increases the electron density and shifts the Fermi level upward. In contrast, RhCl_3_ treatment increases the work function through electron transfer from Ti_3_C_2_T_x_ to Rh^3+^ ions, which are reduced to Rh species on the MXene surface (Figure 4h). The resulting decrease in electron density shifts the Fermi level downward and raises the work function. These results demonstrate that surface molecular adsorption and metal-ion-mediated charge transfer can provide a simple yet effective route for tailoring MXene energy levels, thereby improving charge-selective contact formation in optoelectronic devices such as polymer solar cells and light-emitting diodes [98]. Collectively, the chemical changes induced by surface termination engineering and their corresponding effects on work function modulation, interfacial dipole formation, and energy-level alignment are summarized in Table 3.

## 3. Interfacial Engineering

### 3.1. Surface Modification and Work Function Engineering

Efficient charge injection and extraction in MXene-based optoelectronic devices depend not only on the high conductivity of MXenes but also on their energetic alignment with adjacent semiconductors [99,110]. The chemically accessible surfaces of MXenes enable their work function and interfacial dipoles to be tailored through termination control, molecular adsorption, functionalization, and doping [6,94,112]. These approaches allow Schottky barriers, Fermi-level positions, and charge-selective contacts to be engineered for specific device architectures while retaining the solution processability and metallic transport characteristics of MXenes [92,105,113].

**Figure 5:**
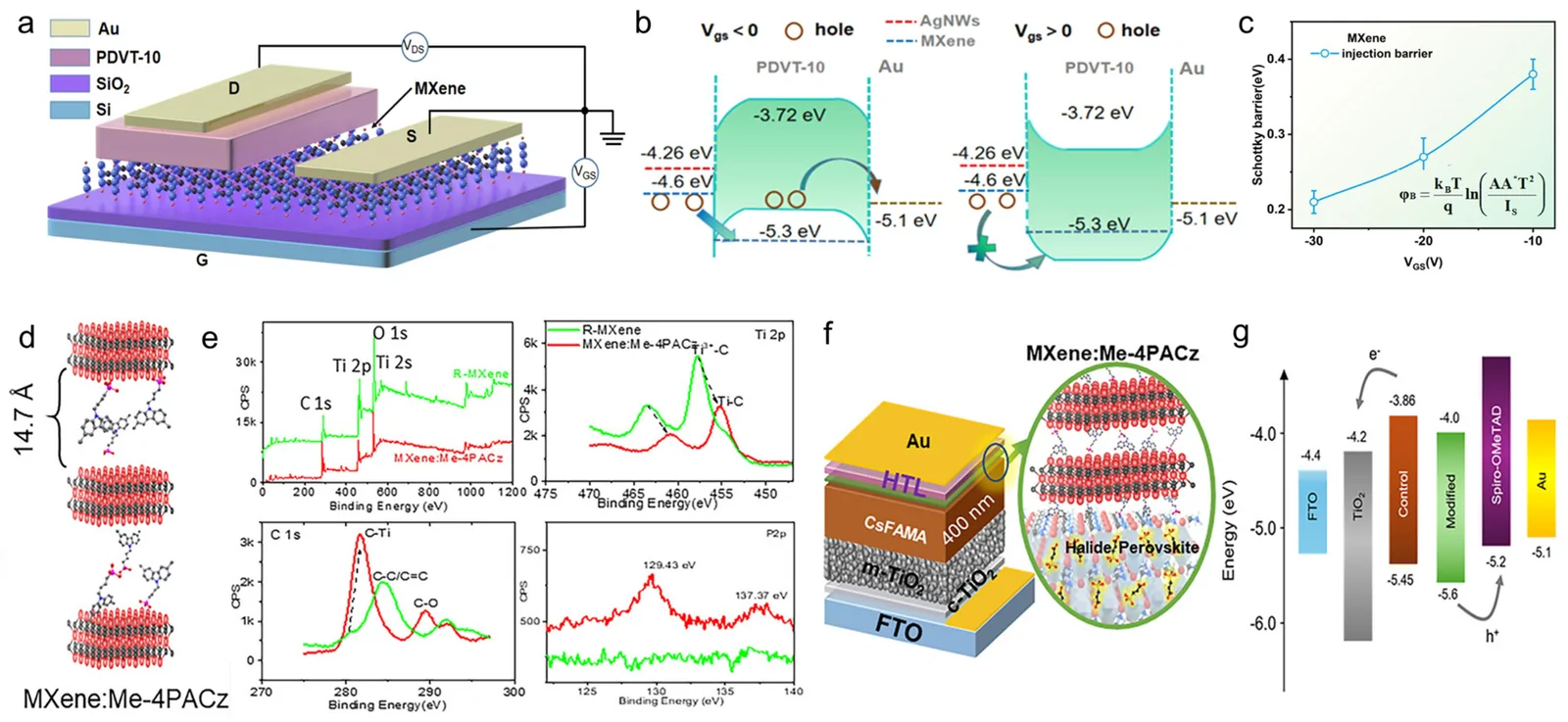
(**a**) Illustration of the MXene-based vertical organic field-effect transistor (MVOFET) device structure. (**b**) Comparative energy band models for MVOFET and AgNWs-based VOFET. (**c**) Gate-bias-dependent variation in the Schottky barrier height in the MVOFET. (**a**–**c**) Reproduced with permission from Ref. [134]. Copyright (2022) Springer Nature. (**d**) Illustrative depiction of Ti_3_C_2_ MXene nanoribbons functionalized with Me-4PACz. (**e**) XPS spectra of Ti 2p, C 1s, and P 2p core levels for delaminated and Me-4PACz-functionalized MXenes. (**f**) PSC device structure and interface detail with Me-4PACz interfacial passivation layer. (**g**) Energy-level alignment of solar cells using modified MXene. (**d**–**g**) Reproduced with permission from Ref. [135]. Copyright (2025) Wiley-VCH GmbH.

Li et al. demonstrated interfacial energy-level control using Ti_3_C_2_T_x_ as the source electrode of a vertical organic field-effect transistor based on p-type PDVT-10 (Figure 5a–c). In such vertical devices, carrier injection is governed primarily by the gate-dependent Schottky barrier at the source/semiconductor interface rather than by conventional channel-charge modulation. The measured Ti_3_C_2_T_x_ work function of approximately 4.6 eV lies closer to the PDVT-10 HOMO level (~5.3 eV) than that of Ag nanowires (~4.26 eV), thereby reducing the intrinsic hole-injection offset (Figure 5b). Temperature-dependent transport analyzed within the thermionic-emission framework showed that the effective barrier decreased from 0.39 to 0.21 eV as V_GS_ changed from −10 to −30 V (Figure 5c). This strong gate dependence arises from the ultrathin, perforated MXene electrode: the gate field directly penetrates the openings while partially transmitting through continuous MXene regions, inducing band bending and hole accumulation in PDVT-10 and consequently lowering and thinning the injection barrier. Together with the smooth MXene/PDVT-10 contact, this dual electrostatic modulation enables efficient carrier injection and a low subthreshold swing of 73 mV/dec, establishing MXene as a gate-tunable injection contact for subsequent interfacial energy-level engineering [134].

Karimipour et al. functionalized delaminated Ti_3_C_2_T_x_ MXene with the carbazole-based phosphonic acid molecule Me-4PACz to tailor its interfacial electronic properties in perovskite solar cells (Figure 5d–g). The incorporation of Me-4PACz increased the MXene interlayer spacing from 9.8 Å in the bulk material to 14.7 Å and produced nanoneedle-like structures approximately 2–3 μm in length and 200–400 nm in diameter (Figure 5d). XPS analysis revealed shifts in the Ti 2p and C 1s components together with the emergence of characteristic P 2p signals, confirming molecular incorporation and substantial modification of the local electronic environment of MXene (Figure 5e). When inserted between the CsFAMA perovskite and Spiro-OMeTAD layers, the MXene:Me-4PACz interlayer shifted the valence- and conduction-band positions from −5.45 and −3.86 eV to −5.60 and −4.00 eV, respectively, producing a more favorable stepwise alignment for hole extraction and suppressing interfacial recombination (Figure 5f,g). Consequently, the modified device achieved a power conversion efficiency of 21.47%, compared with 20.09% for the control, together with an increased open-circuit voltage of 1.16 V and fill factor of 78.12%. The device also retained 88% of its initial maximum-power-point output after 1000 h under ISOS-L-1 testing, whereas the control retained only 45%. These improvements were collectively attributed to optimized energy-level alignment, enhanced charge extraction, reduced deep and shallow trap densities, and increased interfacial hydrophobicity [135].

Additional studies have demonstrated complementary routes for tuning MXene work functions without substantially disrupting their intrinsic surface chemistry. El-Demellawi et al. demonstrated termination-independent work function tuning of Ti_3_C_2_T_x_ using Magic Blue as a noncovalent molecular p-dopant. UPS measurements revealed a stepwise increase in work function from 4.37 to 4.87 eV, arising from two coupled electronic effects. Electron transfer from occupied subsurface states of Ti_3_C_2_T_x_ to the triarylaminium cation shifts the Fermi level downward, while physisorbed SbCl_6^−^_ counterions generate interfacial dipoles with the resulting positive carriers and simultaneously raise the vacuum level. XPS and EPR analyses indicated that the depleted electrons originate predominantly from subsurface graphitic carbon-derived states rather than Ti-related surface states. DFT calculations further confirmed that the Fermi level remains within the density of states after doping, preserving the metallic character of Ti_3_C_2_T_x_. Consistently, transient-absorption measurements showed negligible changes in the surface-plasmon dynamics, supporting a subsurface-doping mechanism that modifies the interfacial energetics without substantially perturbing the native surface terminations or plasmonic response [136].

In a related approach, Nag et al. approached interfacial tuning from the framework of metal–semiconductor junction theory using scanning tunneling spectroscopy together with Schottky-barrier and Fermi-level-pinning analyses. The local *dI*/*dV* spectra, reflecting the electronic density of states, showed that PAH functionalization shifts the characteristic DOS minimum of Ti_3_C_2_T_x_ by approximately −0.24 eV, consistent with electron donation from amine groups and a corresponding decrease in work function. In the conventional Schottky–Mott picture, pristine MXene and *n*-type ZnO exhibit a sizable conduction-band offset, whereas the actual junction behavior is additionally influenced by Fermi-level pinning associated with semiconductor surface states. PAH functionalization shifts the MXene Fermi level closer to the ZnO conduction-band edge, thereby lowering the effective interfacial barrier and weakening the pinning effect. Increased Al doping of ZnO further narrows the depletion region and promotes tunneling through the barrier. Consequently, the PAH·MXene/ZnO junction evolves from rectifying to nearly nonrectifying transport, demonstrating complementary control of barrier height and barrier width for low-resistance electron injection [137].

### 3.2. Heterointerfaces and Barrier Engineering

The performance of MXene-based heterojunctions is strongly governed by interfacial charge redistribution, which determines carrier-transfer pathways, local electric fields, and energy barriers between dissimilar materials [86,95,106,107,109]. Accordingly, molecular passivation and heterostructure construction have been employed to simultaneously stabilize MXene surfaces and regulate their electronic coupling with adjacent optoelectronic components [88,104,106,108,138,139].

**Figure 6:**
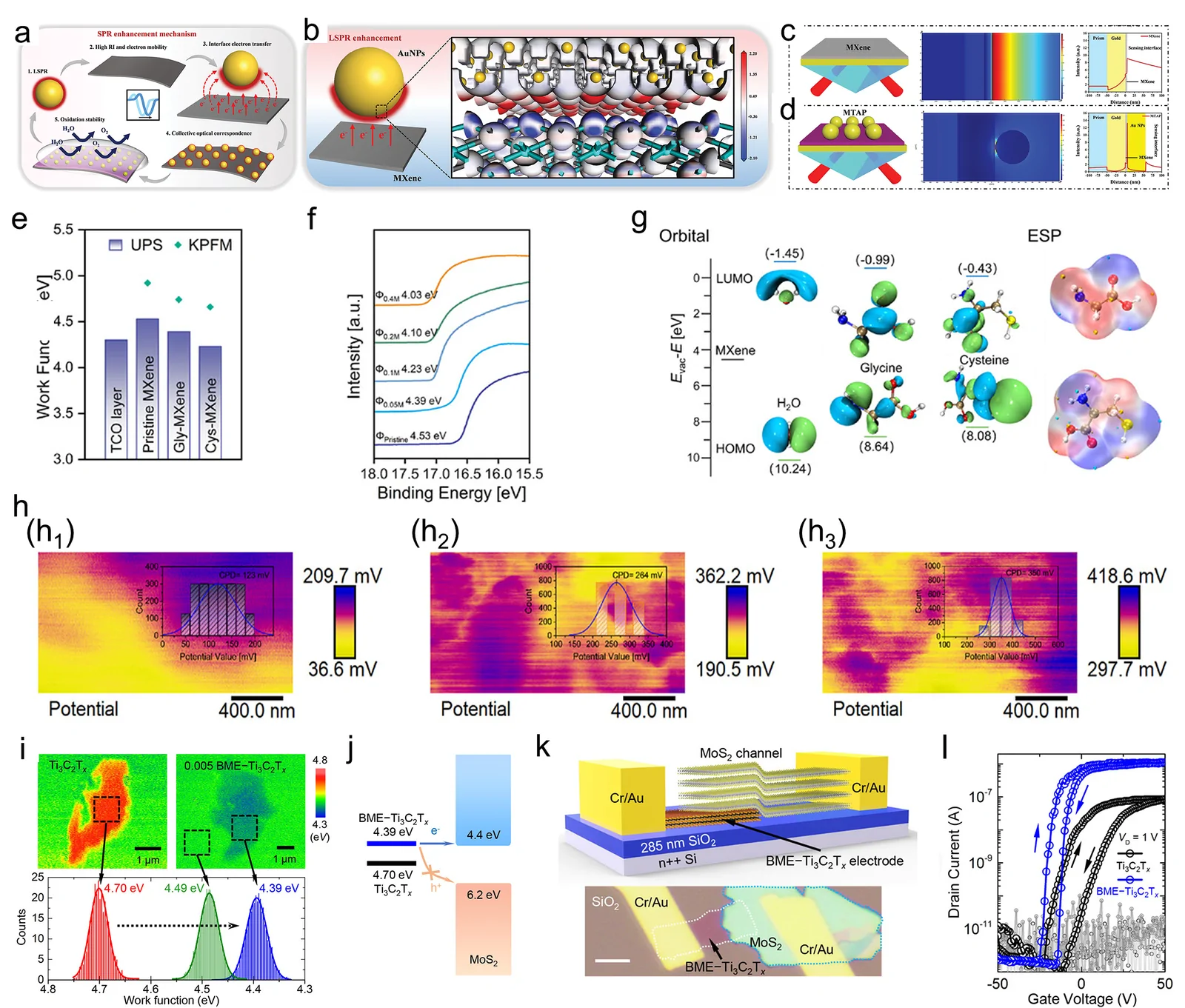
(**a**) Schematic of the SPR enhancement mechanism in the MTAP heterojunction. (**b**) Interfacial charge density difference analysis. FDTD simulations of interfacial electric fields for (**c**) chip-MXene and (**d**) chip-MTAP. (**a**–**d**) Reproduced with permission from Ref. [140]. Copyright (2024) Wiley-VCH GmbH. (**e**) WF determination *via* UPS and KPFM. (**f**) Correlation between the WF shift and the concentration of cysteine doping. (**g**) Orbital energy levels and ESP distributions. (**h**) KPFM-derived CPD maps comparing (**h_1_**) pristine MXene, (**h_2_**) Gly-MXene, and (**h_3_**) Cys-MXene. (**e**–**h**) Reproduced with permission from Ref. [141]. Copyright (2025) Wiley-VCH GmbH. (**i**) Current–voltage characteristics of MoS_2_ field-effect transistors (FETs) utilizing pristine Ti_3_C_2_T_x_ and BME-modified Ti_3_C_2_T_x_ as electrodes. (**j**) Calculated energy band structures for untreated Ti_3_C_2_T_x_, BME-modified Ti_3_C_2_T_x_, and multilayer MoS_2_. (**k**) MoS_2_/BME-MXene FET structure and optical image. (**l**) Transfer characteristics of MoS_2_ FETs with pristine and BME-modified Ti_3_C_2_T_x_ electrodes. (**i**–**l**) Reproduced with permission from Ref. [142]. Copyright (2022) Wiley-VCH GmbH.

Wang et al. demonstrated this concept through a two-dimensional and zero-dimensional heterojunction composed of Ti_3_C_2_T_x_ MXene, tannic acid, Au nanoparticles, and polyethylene glycol, denoted as MTAP (Figure 6a–d). Tannic acid was self-assembled on the MXene surface and used to mediate the in situ growth of Au nanoparticles, while polyethylene glycol occupied the remaining exposed sites and protected the underlying MXene against water- and oxygen-induced degradation. The resulting architecture combined the high refractive index and electron mobility of MXene with the localized surface plasmon resonance of Au nanoparticles, while promoting collective optical coupling among the uniformly distributed nanoparticles (Figure 6a).

At the MXene–Au interface, the work function difference drives electron transfer from MXene to Au nanoparticles. The calculated electron-density difference confirms charge depletion on MXene and accumulation near the Au surface, producing an electron-rich plasmonic interface that strengthens collective carrier oscillation and localized surface plasmon resonance (Figure 6b). Consistent with this interfacial charge redistribution, finite-difference time-domain simulations showed that the maximum electric-field intensity increased from 9.1 for the MXene-modified interface to 13.3 for the MTAP heterojunction (Figure 6c,d). The resulting SPR interface exhibited a refractive-index sensitivity of 176.20°/RIU and enabled label-free exosome detection with a limit of detection of 0.28 particles/mL. Importantly, the tannic acid and polyethylene glycol protective layer also preserved the optical and electrical properties of MXene under oxidative conditions, demonstrating that interfacial charge-transfer engineering can simultaneously enhance plasmonic response and operational stability [140].

Li et al. [141] demonstrated amino acid functionalization as a molecular route for regulating the electronic properties of Ti_3_C_2_T_x_ electrodes in silicon heterojunction solar cells (Figure 6e–h). UPS and KPFM measurements showed that glycine and cysteine reduced the MXene work function from 4.53 eV to 4.39 and 4.23 eV, respectively, improving energy-level matching with ITO (Figure 6e). Increasing the cysteine concentration further enabled continuous work function tuning to 4.03 eV, although excessive molecular loading increased the sheet resistance, indicating a trade-off between energy-level modulation and electrical transport (Figure 6f). Large-area KPFM mapping confirmed that the resulting surface potential shifts were spatially uniform across the functionalized films (Figure 6h).

DFT calculations provided a microscopic basis for this behavior (Figure 6g). Orbital-localization analysis showed that the cysteine HOMO, distributed over the NH_2_ and SH groups, lies closer to the MXene conduction states than that of glycine, favoring stronger wave-function hybridization and electron transfer. Charge density difference calculations further revealed interfacial electron accumulation and depletion consistent with an outward-oriented dipole that lowers the work function. Concurrent Ti–N coordinative and Ti–S covalent bonding stabilizes undercoordinated Ti sites, while molecular dynamics simulations showed reduced water diffusivity and strengthened local Ti–C correlations after functionalization, explaining the improved oxidation resistance. At the device interface, transfer length method analysis showed that H/Ar plasma activation of ITO reduced the MXene/ITO contact resistivity to 0.58 mΩ·cm^2^ by improving wettability, surface carrier density, and conformal contact. This coupled molecular and interfacial engineering enabled spray-coated MXene SHJ cells with efficiencies up to 24.96% and over 90% efficiency retention after 200 days of ambient aging [141].

Jing et al. [142] employed β-mercaptoethanol (BME) treatment to simultaneously stabilize Ti_3_C_2_T_x_ and tune its work function for efficient electron injection into multilayer MoS_2_ (Figure 6i–l). KPFM measurements showed that the work function of Ti_3_C_2_T_x_ decreased from 4.70 to 4.39 eV after treatment with 0.005 M BME, with spatially resolved mapping confirming uniform surface potential modulation across the flakes (Figure 6i). This shift was attributed to electron donation from the sulfhydryl groups, which altered the surface dipole and electronic density of MXene. The resulting work function closely matched the MoS_2_ conduction-band edge of approximately 4.4 eV, thereby minimizing the electron-injection barrier at the MXene/MoS_2_ interface (Figure 6j).

When BME-treated Ti_3_C_2_T_x_ was employed as the source electrode in MoS_2_ field-effect transistors, the contact behavior changed from nonlinear Schottky-type transport to nearly Ohmic transport (Figure 6k,l). Consequently, the ON current increased by approximately one order of magnitude, while an ON/OFF ratio of approximately 10^6^ was achieved. BME also formed strong Ti–S interactions with undercoordinated Ti atoms at MXene edges and defect sites, suppressing oxidative degradation while preserving the two-dimensional morphology and electrical conductivity of Ti_3_C_2_T_x_. This study therefore illustrates that effective heterointerface engineering requires more than energetic matching alone [142].

Work function modulation must be coupled with chemical passivation of electronically active defects, which otherwise act as carrier-trapping and nonradiative recombination centers [107,109]. More broadly, the simultaneous control of energy-level alignment, defect chemistry, and environmental stability provides a rational strategy for reducing charge-transfer losses and establishing reliable MXene interfaces in high-performance optoelectronic devices [88,138,139].

### 3.3. Interfacial Defect Passivation

Beyond energy-level alignment, the passivation of interfacial defects is essential for suppressing carrier trapping and nonradiative recombination in optoelectronic devices [139]. Owing to their termination-rich and chemically active surfaces, MXenes can coordinate with undercoordinated atoms, regulate defect states, and simultaneously improve interfacial charge transport [6,99,107]. This subsection highlights representative studies in which chemically modified MXenes were employed to passivate semiconductor interfaces and thereby enhance carrier dynamics, device efficiency, and operational stability.

Yao et al. treated Ti_3_C_2_T_x_ with SnCl_4_ to passivate the defect-rich interface in pyramid-textured *n*-Si/MXene Schottky solar cells. The optimized treatment reduced the reverse saturation current density and diode ideality factor, increased the recombination resistance, and prolonged the minority-carrier lifetime from 6.29 to 19.06 μs. These improvements suppressed defect-assisted recombination and facilitated charge extraction, increasing the device efficiency from 4.7% to 9.3% while retaining approximately 88% of the initial performance after 30 days in ambient air [143]. Similarly, Wu et al. introduced Ti_2_CT_x_ as an interfacial passivation layer in inverted perovskite solar cells, where its surface terminations simultaneously regulated defect chemistry, crystallization, and band alignment. XPS and FTIR analyses supported a Lewis acid–base interaction between O-containing MXene terminations and undercoordinated Pb^2+^ sites, forming Ti–O–Pb linkages that suppress deep traps and nonradiative recombination. Consistent with this mechanism, space-charge-limited-current analysis showed lower trap-filled limit voltages, while carrier mobilities extracted using the Mott–Gurney model increased, particularly for electrons. GIWAXS further revealed preferential growth along the (001) direction, indicating that MXene-assisted recrystallization promotes larger, vertically oriented grains with fewer grain-boundary defects. UPS measurements showed that the perovskite work function decreased from 4.81 to 4.52 eV and the Fermi level shifted toward the conduction band, producing more favorable alignment with PCBM and facilitating electron extraction. These coupled effects increased the PCE from 17.95% to 22.22%, while the modified devices retained over 80% of their initial efficiency after 1200 h under ambient conditions [144].

**Figure 7:**
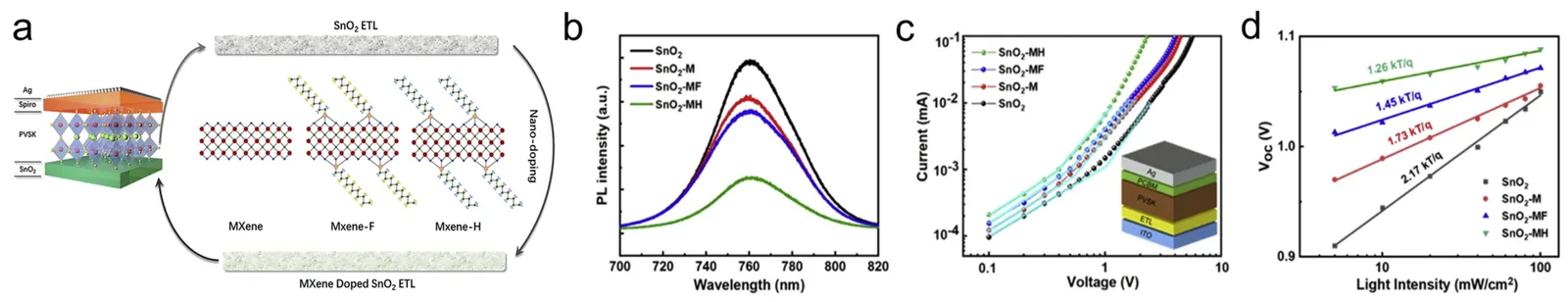
(**a**) Schematics of device structure and crystal structures of MXene, MXene-F, and MXene-H. (**b**) Steady-state PL spectra of perovskite thin films fabricated on various ETLs. (**c**) SCLC characterization of electron-only devices using different ETLs in an ITO/ETL/PVSK/PCBM/Ag structure. (**d**) Dependence of *V_oc_* on incident light intensity for investigating recombination mechanisms. (**a**–**d**) Reproduced with permission from Ref. [145]. Copyright (2022) Elsevier Inc.

Yin et al. incorporated dodecyltrimethoxysilane-functionalized Ti_3_C_2_T_x_, denoted as MXene-H, into a SnO_2_ electron-transport layer to simultaneously regulate interfacial defects, energy-level alignment, and electron transport (Figure 7a–d). Organosilane functionalization reduced OH-related trap states in SnO_2_ and adjusted its surface energy and work function, while conductive MXene nanosheets formed nanoscale heterojunctions that facilitated electron transfer (Figure 7a). The SnO_2_-MH interface exhibited favorable band alignment and a near-zero Schottky barrier, thereby promoting charge extraction from the perovskite absorber.

Consistently, SnO_2_-MH produced the strongest photoluminescence quenching and reduced the perovskite trap density from 3.21 × 10^16^ to 9.93 × 10^15^ cm^−3^ (Figure 7b,c). The diode ideality factor also decreased from 2.17 to 1.26, indicating substantial suppression of trap-assisted recombination (Figure 7d). Consequently, the champion device efficiency increased from 20.98% to 24.12%, while approximately 80% of the initial efficiency was retained after 1000 h of continuous illumination. These results demonstrate that functionalized MXene dopants can concurrently passivate ETL defects, optimize interfacial energetics, and accelerate charge transport in perovskite optoelectronic devices [145].

Collectively, the findings reviewed in Section 3 underscore interfacial engineering as a unifying framework through which the intrinsic physicochemical attributes of MXenes are translated into tangible optoelectronic device performance [102,138]. Beyond their well-established role as conductive interlayers, MXenes demonstrate a remarkable capacity to govern interfacial energetics, modulate charge-transfer pathways, passivate defect states, and reinforce chemical stability-capabilities that arise directly from the compositional versatility and surface-chemical tunability inherent to this materials class [138,139]. The concerted application of work function engineering, heterojunction optimization, and defect passivation yields interfaces that simultaneously promote selective carrier transport and suppress both nonradiative recombination and long-term degradation [6,86,103,106]. Taken together, these attributes position MXenes as multifunctional interfacial materials uniquely suited for the rational integration of electrodes, carrier-transport layers, and photoactive components within high-performance, durable optoelectronic architectures [6,105,113]. The principal interfacial engineering strategies discussed herein, together with their respective functional roles and the associated device-level improvements, are consolidated in Table 4.

## 4. Optoelectronic Devices

### 4.1. Transparent Conducting Electrodes (TCEs)

Transparent conducting electrodes are increasingly expected to provide functions beyond optical transmission and electrical conduction, including solar energy harvesting [6,146], Joule heating [1,5], thermal management [1], and mechanical actuation [146]. MXenes are attractive for these multifunctional platforms because their conductivity, transparency, and mechanical compliance can be tuned through film thickness, interlayer chemistry, surface structuring, and hybridization with conductive nanomaterials [1,2,146]. This subsection highlights representative studies in which MXene-based transparent electrodes were structurally engineered to improve interflake transport, optical absorption, and thermal conversion while retaining sufficient transparency for optoelectronic integration. Their structural characteristics, electrical and optical properties, and representative applications are summarized in Table 5.

Li et al. [146] approached the transparency–conductivity trade-off from a different direction, wrinkling a Ti_3_C_2_T_x_/Ag nanowire film at two length scales rather than optimizing flake alignment. Hydrogen bonding between the two components drove the assembly of a primary wrinkled network; a subsequent protic acid treatment exchanged intercalated Li^+^ for H^+^, contracting the MXene interlayer spacing and folding a finer set of secondary wrinkles into the existing corrugation. The texture mattered optically. At a 3:1 MXene-to-nanowire ratio, the film transmitted roughly 74% of visible light while reflecting less of it, since the corrugated surface trapped incident photons that a flat electrode would have turned away, and charge transport improved at the same time. Both effects were exploited in a PI/LDPE bimorph actuator, where one-sun illumination raised the film temperature by about 40 °C and bent the device to a curvature above 7 cm^−1^. The same film reached approximately 95 °C under 1 V as a Joule heater, and its thermal response survived repeated bending and extended storage in ambient air. Surface architecture and interlayer ion chemistry, in other words, need not be pursued separately; here they were tuned together to yield an electrode that heats efficiently while remaining see-through [146].

**Figure 8:**
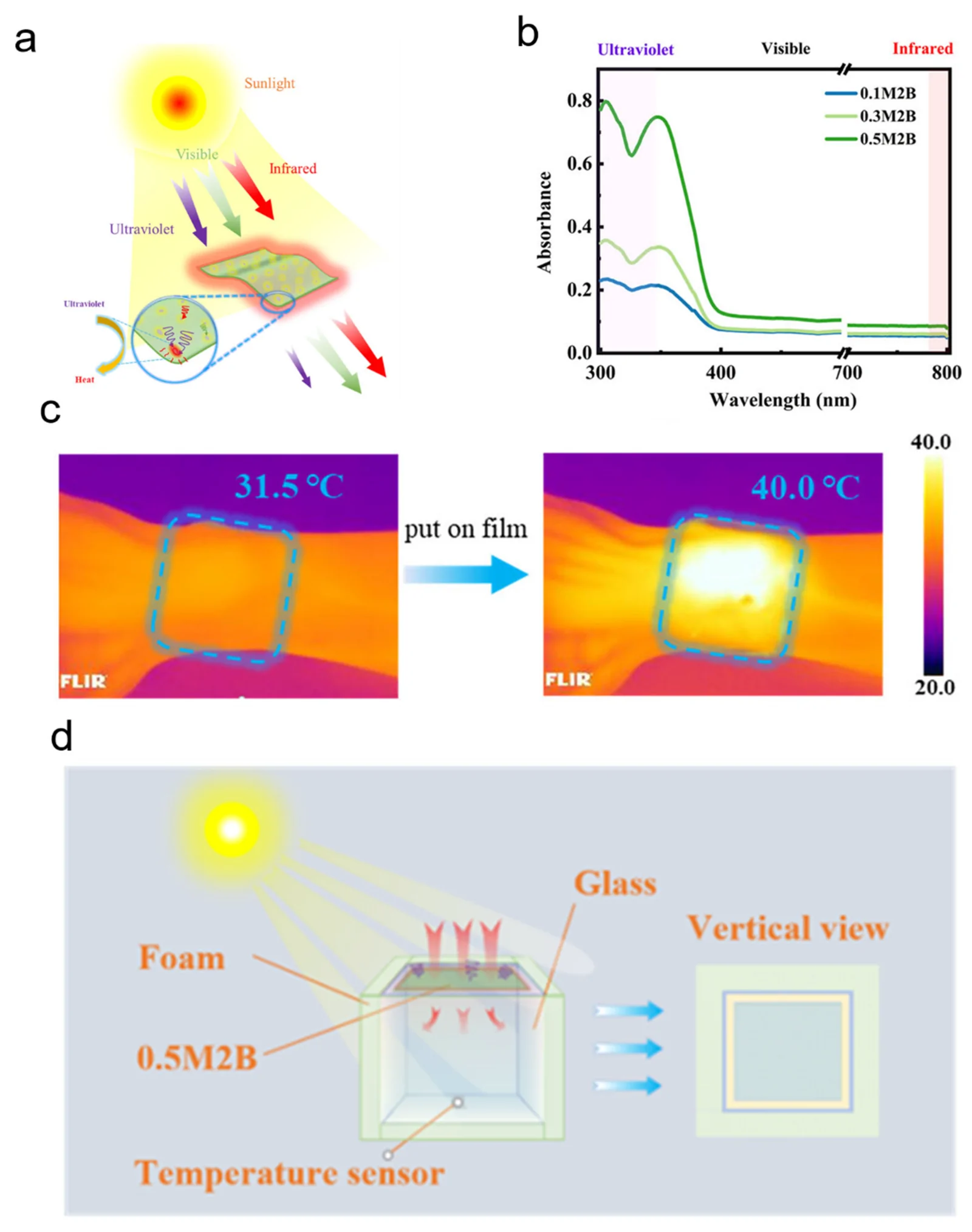
(**a**) Visualization of the photothermal conversion process of a UHMWPE composite film. (**b**) Absorption spectra of UHMWPE films with BZT and various MXene contents. (**c**) Infrared (IR) thermal image of the wrist and the attached film following 1 min of irradiation. (**d**) Schematic diagram of an outdoor cooling performance measurement system. Reproduced with permission from Ref. [150]. Copyright (2024) Springer Nature.

Liu et al. [150] developed transparent MXene@BZT/UHMWPE composite films that combine selective ultraviolet absorption with photothermal conversion and solar heat-management capabilities (Figure 8a–d). The benzotriazole-based UV absorber BZT formed hydrogen-bonding interactions with Ti_3_C_2_T_x_ and improved its dispersion within the UHMWPE matrix, thereby suppressing light-scattering agglomerates while maintaining visible-light transmittance above 85% and haze below 12%. As illustrated in Figure 8a, the composite selectively absorbs ultraviolet radiation below 400 nm while transmitting most visible light, and the absorbed optical energy is subsequently converted into heat by the dispersed MXene nanosheets.

The absorbance spectra show that increasing the MXene content substantially strengthens absorption in the ultraviolet region while producing only a modest increase across the visible and near-infrared ranges (Figure 8b). This spectral selectivity arises from the strong UV absorption of the triazole and hindered phenol groups in BZT, together with contributions from TiO_2_-related species formed by partial MXene oxidation. BZT itself contributed negligibly to photothermal heating, whereas its dispersion-promoting effect enhanced the light-to-heat conversion of MXene. Accordingly, a 0.5M2B film placed on the wrist reached 40.0 °C after 60 s of irradiation at 100 mW/cm^2^, compared with a surrounding skin temperature of 31.5 °C (Figure 8c).

The film was further evaluated as a transparent window covering using an outdoor glass-container model (Figure 8d). Complete coverage with the 0.5M2B film reduced the internal temperature by approximately 6–7 °C relative to uncovered glass while preserving more than 80% of the incident indoor illumination. Building energy simulations further projected annual cooling energy savings of approximately 31–61 MJ/m^2^ across representative climatic regions, corresponding to reductions of up to approximately 12% in cooling energy consumption. These results demonstrate that controlled MXene dispersion within transparent polymer matrices can reconcile UV shielding, photothermal functionality, optical clarity, and passive suppression of solar heat gain [150].

### 4.2. Solar Cells

MXenes have emerged as versatile interfacial materials for solar cells because their high conductivity [63], tunable work function [47,65,74,75], and termination-rich surfaces enable simultaneous control over film formation, charge extraction, and interfacial recombination. In perovskite and other thin-film photovoltaic systems, they have been incorporated as interlayers [12,13], transport-layer additives [9,10], and electrodes [8] to improve energy-level alignment [7,8,9,10,12], passivate defects [7,10,12], and balance electrical transport with optical transparency [7,8]. This subsection highlights representative MXene-enabled solar-cell architectures and their interfacial roles, while their device structures and photovoltaic performances are summarized in Table 6.

Yuan et al. introduced a Ti_3_C_2_T_x_ interlayer between SnO_2_ and the perovskite absorber in semi-transparent perovskite solar cells. Interactions between the MXene surface terminations and perovskite precursor species moderated crystallization, increased the average grain size from 174 to 265 nm, and reduced grain-boundary-related recombination. At an optimized MXene concentration of 1 mg/mL, the short-circuit current density increased from 17.56 to 20.11 mA/cm^2^ and the power conversion efficiency improved from 12.37% to 14.78%. The resulting semi-transparent device exhibited an average visible transmittance of 22.68% over 400–760 nm, or 26.7% over 400–800 nm, and retained 85.58% of its initial efficiency after more than 1000 h under ambient conditions. These results demonstrate that controlled MXene interlayers can simultaneously improve perovskite morphology, charge extraction, optical transparency, and operational stability, providing an effective interface engineering strategy for building-integrated photovoltaics and power-generating windows [153].

**Figure 9:**
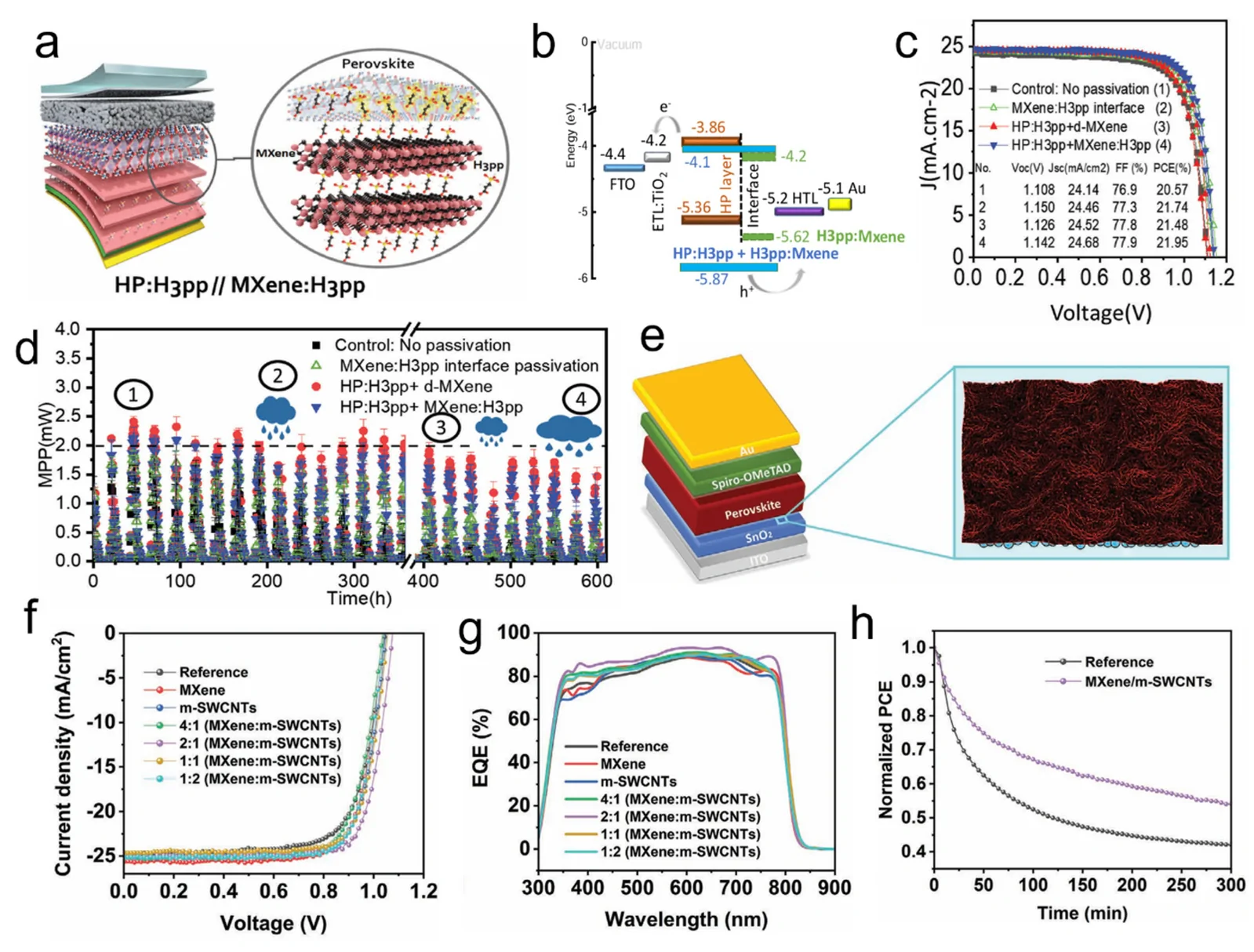
(**a**) PSC structure using H3pp ligand-treated HP and MXene. (**b**) Energy band diagram of PSCs using HP:H3pp + MXene:H3pp and MXene:H3pp. (**c**) *J-V* curves of four PSC configurations with different MXene and H3pp passivation conditions. (**d**) Average MPP values of four device configurations according to ISOS-O protocol. Reproduced with permission from Ref. [152]. Copyright (2023) Wiley-VCH GmbH. (**e**) PSC device structure with an MXene/m-SWCNT interfacial layer on SnO_2_. (**f**) *J-V* characteristics of MXene/m-SWCNT devices according to MXene/m-SWCNT ratio. (**g**) EQE spectra of MXene/m-SWCNT devices according to MXene/m-SWCNT ratio. (**h**) Efficiency of devices with and without an MXene/m-SWCNTs hybrid interface layer upon illumination. Reproduced with permission from Ref. [155]. Copyright (2021) Wiley-VCH GmbH.

Karimipour et al. developed a dual-passivation strategy in which 3-phosphonopropionic acid (H3pp) was incorporated into both the halide perovskite absorber and the Ti_3_C_2_T_x_ MXene interlayer (Figure 9a–d). The resulting HP:H3pp/MXene:H3pp heterojunction created a chemically connected interface between the perovskite and Spiro-OMeTAD, where H3pp passivated perovskite defects and suppressed ion migration, while Ti–O–P bonding stabilized the functionalized MXene (Figure 9a). The dual modification also shifted the interfacial valence-band position to approximately −5.87 eV, providing favorable energetic coupling for hole extraction and reducing interfacial recombination (Figure 9b).

Consequently, the dual-passivated device achieved a power conversion efficiency of 21.95%, exceeding those of the control and singly modified devices (Figure 9c). More importantly, it exhibited superior stability under real outdoor ISOS-O conditions, maintaining its photovoltaic response during variations in irradiation, humidity, and rainfall and showing pronounced recovery after low-light or rainy periods (Figure 9d). A T_80_ lifetime of approximately 600 h was obtained, whereas the control device degraded substantially earlier. These findings demonstrate that coordinated passivation of both the perovskite bulk and MXene interface can simultaneously regulate energy-level alignment, defect states, ion migration, and environmental stability in perovskite solar cells [152].

Bati et al. introduced a one-dimensional and two-dimensional hybrid interlayer composed of metallic single-walled carbon nanotubes and Ti_3_C_2_T_x_ MXene between SnO_2_ and the perovskite absorber (Figure 9e–h). The metallic nanotubes formed conductive pathways between MXene nanosheets, compensating for the conductivity loss associated with partial MXene oxidation during UV–ozone treatment, while the modified interface reduced oxygen-vacancy-related defects and facilitated electron extraction (Figure 9e). The photovoltaic response strongly depended on the hybrid composition, with an optimized MXene to m-SWCNT mass ratio of 2:1 providing the most favorable balance between electrical transport and interfacial recombination. Excessive nanotube loading instead increased recombination because of the ambipolar and gapless metallic characteristics of m-SWCNTs.

The optimized hybrid device exhibited enhanced current–voltage and external quantum efficiency characteristics, achieving a short-circuit current density of 25.09 mA/cm^2^, an open-circuit voltage of 1.07 V, a fill factor of 0.80, and a power conversion efficiency of 21.42% (Figure 9f,g). The improvement was primarily associated with faster electron extraction, reduced interfacial charge-transfer resistance, and suppressed trap-assisted recombination. Under continuous one-sun illumination, the hybrid device retained approximately 55% of its initial efficiency after 300 min, compared with approximately 40% for the reference device (Figure 9h). This enhanced photostability was attributed to interfacial defect passivation and potentially improved heat dissipation through the nanotube network. These results demonstrate that hybridizing MXene with conductive one-dimensional nanomaterials can mitigate oxidation-induced transport losses while improving interfacial charge dynamics in perovskite solar cells. More broadly, these studies establish MXene as a multifunctional interfacial component that can simultaneously regulate charge transport, defect passivation, energy-level alignment, and device stability in diverse photovoltaic architectures. Such capabilities highlight the potential of rationally engineered MXene interfaces to bridge the remaining gap between high laboratory efficiency and reliable, scalable solar-cell operation [155].

### 4.3. Photodetectors

MXenes offer a distinctive combination of metallic conductivity [64], solution processability [51], optical transparency [48,50], and tunable work function [54,76,77], making them attractive electrodes and interfacial layers for photodetectors. When coupled with semiconductors, MXenes can form Schottky junctions whose built-in electric fields promote carrier separation and collection without an external bias [25]. Their optical and electronic properties can also be regulated through film thickness, surface chemistry, and heterojunction design [15], enabling self-powered photodetection across ultraviolet, visible, and infrared spectral regions. Recent MXene-based photodetectors, their device configurations, and key performance characteristics are summarized in Table 7.

**Figure 10:**
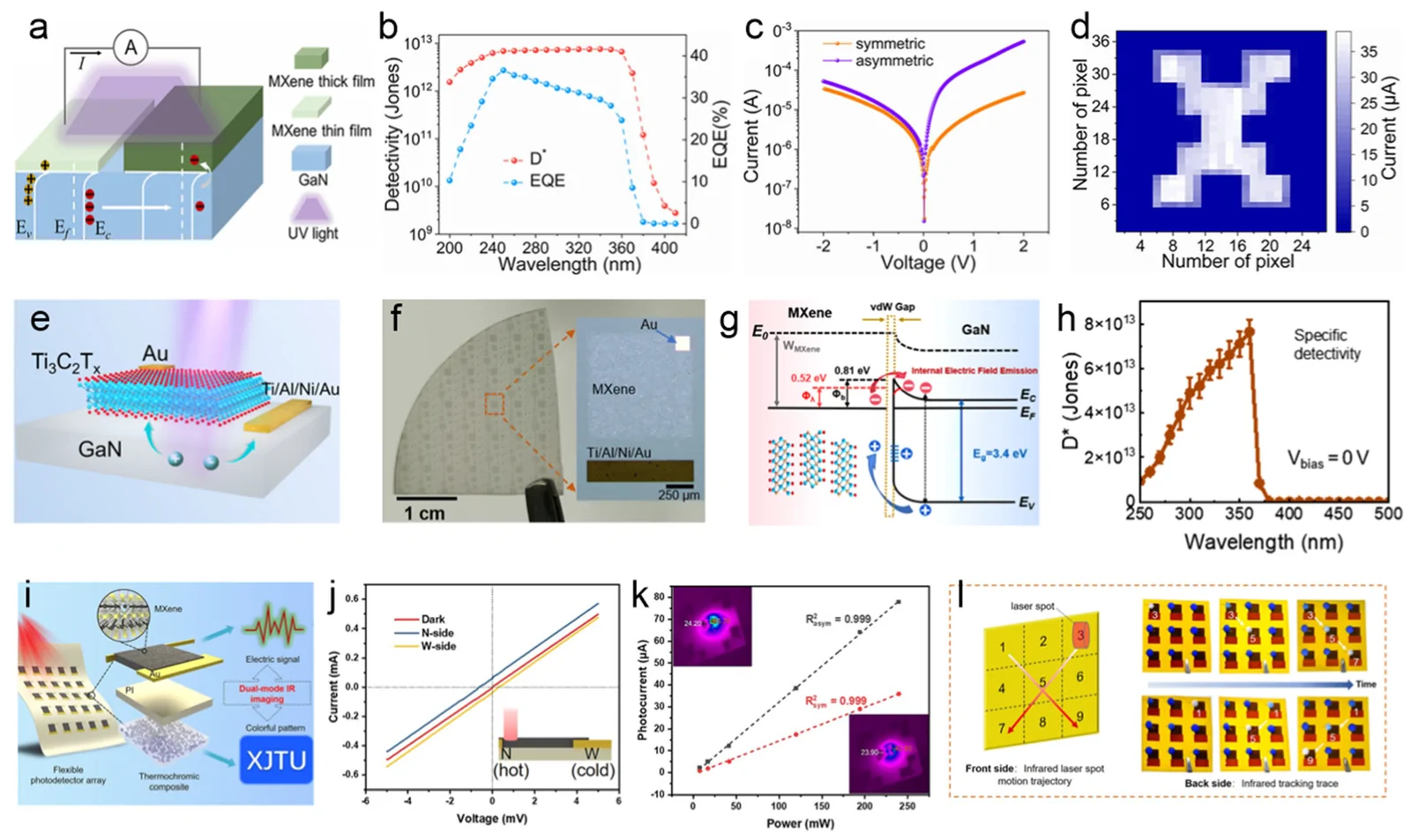
(**a**) Structure and charge transport processes of MXene/GaN films under UV irradiation. (**b**) EQE and D* of MXene/GaN photodetector evaluated under no-voltage conditions. (**c**) *I-V* curves with and without electrode transmittance contrast. (**d**) “X” character obtained by applying MXene/GaN to UV imaging. Reproduced with permission from Ref. [160]. Copyright (2024) John Wiley and Sons. (**e**) Device structure of a UV photodetector. (**f**) Device fabricated on a GaN substrate. (**g**) Energy band profile of MXene/GaN Schottky heterostructure under dark and UV irradiation conditions. (**h**) Specific detectivity of the photodetector at 0 V bias. (7.65 × 10^13^ Jones). Reproduced with permission from Ref. [162]. Copyright (2024) AIP Publishing. (**i**) Configuration of a dual-mode (human-machine) infrared-detectable photodetector array. (**j**) *I-V* curves induced by the Seebeck effect in an asymmetric PTE photodetector. (**k**) Photocurrent and infrared thermal images of symmetric and asymmetric devices upon irradiation at 0 V bias. (**l**) Applications for infrared trajectory tracking. Reproduced with permission from Ref. [159]. Copyright (2025) Springer Nature.

Ma et al. demonstrated a self-powered ultraviolet photodetector based on transmittance contrast between two Ti_3_C_2_T_x_ electrodes deposited on GaN (Figure 10a–d). The thin and thick MXene electrodes had thicknesses of approximately 44 and 126 nm, respectively, producing an average transmittance contrast of approximately 84%, while maintaining similar work functions of 4.58 and 4.54 eV. Because these values exceed the GaN work function of approximately 4.0 eV, Schottky junctions and built-in electric fields were formed at both MXene/GaN interfaces. Under UV illumination, photocarriers were generated primarily beneath the transparent MXene electrode, whereas the opaque electrode suppressed light absorption in the underlying GaN. This asymmetric carrier generation induced lateral electron diffusion and produced a net photocurrent at zero bias (Figure 10a).

The resulting device exhibited a peak responsivity of 81 mA/W, rise and decay times below 31 and 29 ms, respectively, and an on/off ratio of 1.33 × 10^6^. Spectral measurements further revealed a maximum external quantum efficiency of 36.6% at 250 nm and a specific detectivity of 7.57 × 10^12^ Jones at 340 nm, while the wide bandgap of GaN provided intrinsic visible-blind detection (Figure 10b). In contrast, devices employing two equally transparent MXene electrodes produced nearly cancelling photocurrents under uniform illumination, confirming that electrode transmittance asymmetry was essential for self-powered operation (Figure 10c). The photodetector also reproduced a well-resolved X-shaped ultraviolet image containing 1026 pixels, demonstrating its potential for self-powered imaging applications (Figure 10d). These findings establish thickness-controlled MXene electrodes as a simple solution-processed platform for generating optical and electrical asymmetry without employing dissimilar electrode materials [160].

Ding et al. developed a self-powered ultraviolet photodetector based on an asymmetric Ti_3_C_2_T_x_ MXene/GaN van der Waals Schottky junction (Figure 10e–h). A solution-processed MXene film formed a Schottky contact with GaN, whereas the Ti/Al/Ni/Au electrode provided an Ohmic contact, generating an asymmetric built-in electric field for carrier separation at zero bias (Figure 10e,f). The MXene film exhibited approximately 72.9% transmittance at 360 nm and formed a clean vdW interface with GaN, thereby reducing process-induced defect states while enabling efficient carrier collection.

The average Schottky barrier height was approximately 0.81 eV, and UV illumination further lowered the effective barrier through surface-state-mediated hole trapping, contributing to photocurrent gain (Figure 10g). Consequently, the device achieved a responsivity of 681.6 mA/W, an EQE of 234.7%, and a specific detectivity of 7.65 × 10^13^ Jones at 360 nm under zero bias (Figure 10h). The detector retained approximately 96.3% of its initial response after three months and about 90% after twelve months, demonstrating that high-quality MXene/GaN vdW interfaces can simultaneously support efficient self-powered detection and long-term operational stability [162].

Fang et al. developed a flexible dual-mode infrared photodetector by integrating a Ti_3_C_2_T_x_ MXene-based photothermoelectric device with a thermochromic composite (Figure 10i–l). Under 808 nm illumination, the strong photothermal conversion of MXene generated a local temperature gradient that drove carrier transport through the Seebeck effect, enabling self-powered electrical detection. The linear current–voltage characteristics confirmed Ohmic contact between the MXene film and Au electrodes, while the finite short-circuit current at zero bias verified photothermoelectric operation (Figure 10j).

Geometrically asymmetric Au electrodes further enhanced the thermal gradient by suppressing heat dissipation at the narrow electrode. The asymmetric device reached a hot-side temperature of 85.7 °C while maintaining the opposite electrode at 24.2 °C, compared with a temperature difference of only 18.9 °C in the symmetric configuration (Figure 10k). Consequently, the responsivity increased from 0.15 to 0.33 mA/W. The device also maintained nearly unchanged electrical and photoresponse characteristics after 300 bending cycles at 0.31% strain, reflecting the flexibility and low resistance of the MXene film.

By coupling MXene-mediated heat generation with a thermochromic composite that changed from blue to white, invisible infrared radiation was additionally converted into a human-readable optical pattern. The array simultaneously produced machine-readable electrical signals and visually traceable color changes, enabling both static imaging and dynamic tracking of infrared trajectories such as 3→5→7 and 1→5→9 (Figure 10l). This dual-output architecture demonstrates how MXene photothermal and thermoelectric properties can be integrated to realize flexible human–machine collaborative infrared sensing. Overall, these studies establish MXenes as versatile components for photodetectors, where their electrical, optical, and photothermal properties can be tailored to enable self-powered detection across ultraviolet and infrared spectral regions. Continued integration of MXenes with semiconductor junctions and functional optical materials is expected to advance photodetectors toward flexible, energy-efficient, and intelligent imaging systems [159].

### 4.4. Light-Emitting Devices (LEDs)

MXenes are increasingly employed in light-emitting devices as flexible transparent electrodes and interfacial components because they combine high conductivity, mechanical compliance [43,49], solution processability, and chemically active surfaces. In particular, integration with metallic nanowire networks can reduce junction resistance, improve substrate adhesion, and mitigate the mechanical brittleness associated with conventional ITO electrodes [3,4]. Recent MXene-based light-emitting-device architectures, electrode-engineering approaches, and electroluminescence characteristics are summarized in Table 8.

**Figure 11:**
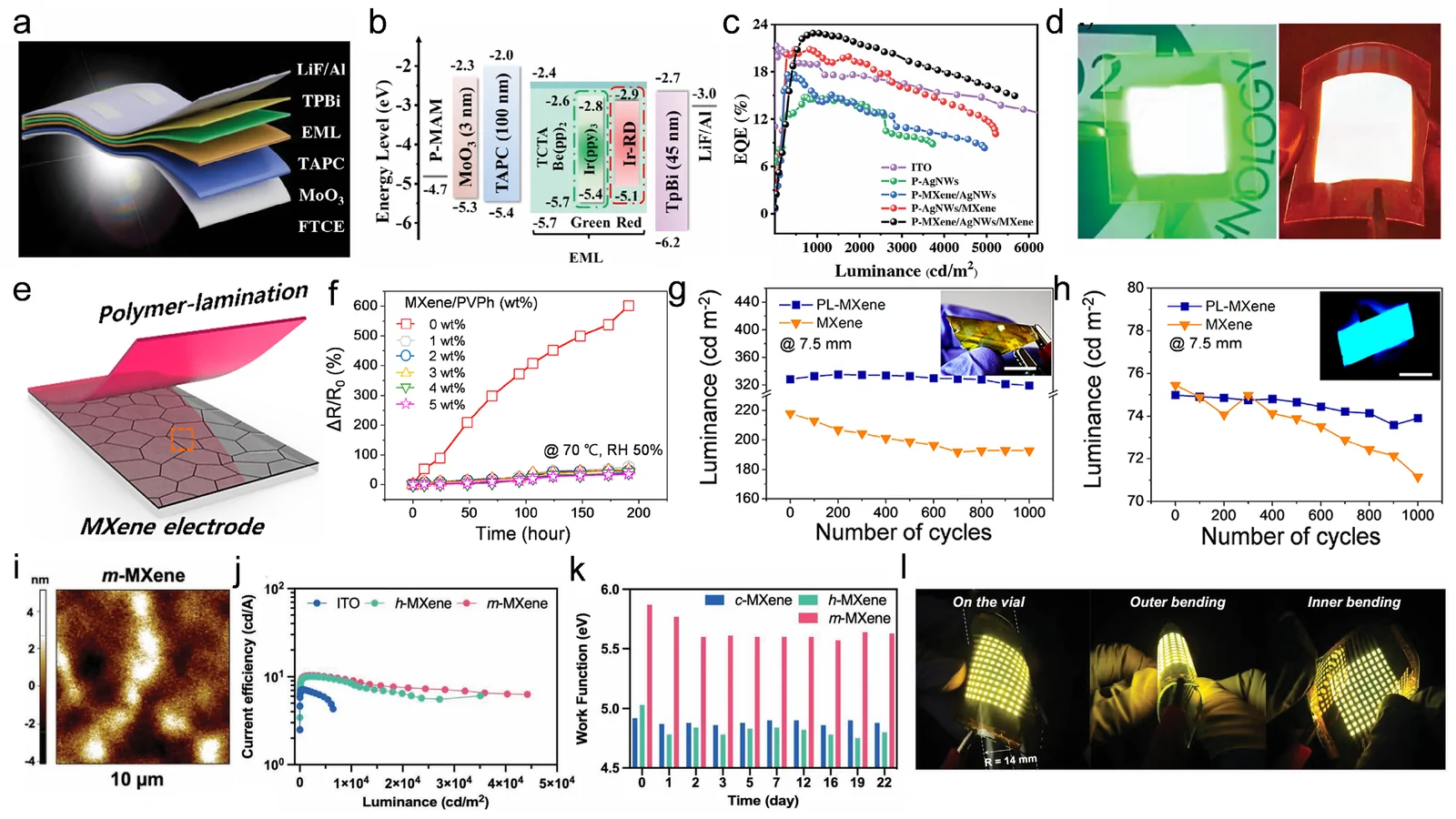
(**a**) FTCE-based OLED device structure. (**b**) Energy-level diagrams of FTCE-based green and red OLEDs. (**c**) EQE-L curves of FTCE-based green OLEDs. (**d**) Emission photographs of FTCE-based green and red OLEDs. (25 × 25 mm^2^) Reproduced with permission from Ref. [167]. Copyright (2025) Wiley-VCH GmbH. (**e**) PL-MXene electrode structure composed of MXene flakes covered with PVPh polymer. (**f**) Resistance change in PL-MXene showing high oxidation stability under conditions of 70 °C and 50% humidity. (**g**) Change in luminance with bending cycles of bare MXene and PL-MXene-based organic AC-EL displays (inset: photograph of a PL-MXene-based organic AC-EL display). (**h**) Change in luminance with bending cycles of bare MXene and PL-MXene-based inorganic AC-EL displays (Inset: photograph of a PL-MXene-based inorganic AC-EL display). Reproduced with permission from Ref. [171]. Copyright (2021) American Chemical Society. (**i**) AFM topographic visualization of m-MXene within a 10 µm scan area. (**j**) OLED current efficiency. (**k**) Change in work function for three types of MXene at room temperature and relative humidity of approximately 40%. (**l**) OLED light emission on a 6 cm × 6 cm substrate with electrodes patterned using photoresist. Reproduced with permission from Ref. [170]. Copyright (2022) Wiley-VCH GmbH.

Yang et al. [167] developed a pressure-treated Ti_3_C_2_T_x_/Ag nanowire/Ti_3_C_2_T_x_ sandwich electrode for high-performance flexible OLEDs (Figure 11a–d). The MXene layers enclosed the AgNW network and promoted intimate contact at wire-to-wire junctions, while mechanical pressing further embedded the nanowires into the PET substrate. This architecture reduced the sheet resistance to 20.5 Ω/sq while maintaining a transmittance of 92.3% at 550 nm. Capillary forces generated during deposition of the upper MXene layer drew nanosheets toward AgNW intersections, increasing conductive pathways and reducing the surface roughness to approximately 8.0 nm. Hydrogen-bonding interactions between MXene surface terminations and the hydrophilic PET substrate additionally enhanced electrode adhesion and mechanical durability.

The sandwich electrode was subsequently incorporated as the anode in green and red phosphorescent OLEDs with MoO_3_ and LiF injection layers and TAPC and TPBi transport layers (Figure 11a,b). The favorable conductivity, smooth morphology, and balanced carrier injection of the hybrid electrode enabled the green OLED to achieve a maximum external quantum efficiency of 22.9% and a current efficiency of 81.4 cd/A, exceeding the corresponding ITO-based reference performance (Figure 11c). The red device exhibited a maximum EQE of 24.6% with limited efficiency roll-off, while both devices produced bright and spatially uniform emission from flexible 25 × 25 mm^2^ panels (Figure 11d). These results demonstrate that MXene/AgNW hybridization can translate nanoscale junction welding and interfacial adhesion into efficient, mechanically robust, and large-area flexible light-emitting devices [167].

Lee et al. [171] introduced a polymer-laminated MXene electrode by coating a thin, cross-linked poly(4-vinylphenol) layer onto a solution-processed Ti_3_C_2_T_x_ film (Figure 11e–h). Polar interactions between the hydroxyl groups of PVPh and the termination-rich MXene surface enabled the formation of a uniform protective layer without substantially compromising the conductivity, transparency, or structural ordering of the underlying electrode (Figure 11e). Under accelerated aging at 70 °C and 50% relative humidity, the resistance of bare MXene increased by approximately 600% after 200 h, whereas PVPh-laminated electrodes exhibited considerably smaller changes of approximately 35–60%, confirming effective suppression of moisture- and oxygen-induced oxidation (Figure 11f).

The insulating PVPh layer further enabled the PL-MXene electrode to function in field-driven organic and inorganic AC electroluminescent displays. An organic Super Yellow-based device retained stable light emission after 1000 bending cycles at a radius of 7.5 mm, with substantially less luminance degradation than the device employing bare MXene (Figure 11g). Similarly, the ZnS:Cu/PDMS inorganic AC-EL display preserved its emission under both repeated bending and harsh environmental exposure (Figure 11h). These results demonstrate that polymer lamination can simultaneously improve the oxidation resistance and mechanical reliability of transparent MXene electrodes, extending their applicability to flexible and environmentally robust light-emitting devices [171].

Beyond electrode modification, Jeong et al. [168] demonstrated that the environmental reliability of MXene-based OLEDs could be further improved through a stress-balanced bilayer encapsulation composed of an Al_2_O_3_/TiO_2_ nanolaminate and an S–H nanocomposite polymer. The encapsulated RGB OLEDs exhibited luminance exceeding 1000 cd/m^2^ and retained approximately 70% of their initial luminance after 2040 h in air, while maintaining stable operation after 5000 bending cycles at a radius of 1.5 mm and 6 h of water immersion. This study highlights that integrating moisture-barrier design with mechanical-stress compensation is essential for translating MXene transparent electrodes into reliable foldable and water-resistant displays [168].

Additionally, Zhou et al. [170] developed a surface-modified Ti_3_C_2_T_x_ electrode, denoted as m-MXene, by combining thermal densification with a perfluorosulfonic acid barrier layer (Figure 11i–l). Thermal annealing removed intercalated water and residual surface species, reduced the interlayer spacing, and improved interflake contact, thereby producing a highly compact h-MXene film. Subsequent PFSA modification uniformly covered the electrode surface and passivated gaps between adjacent flakes, as evidenced by the disappearance of individual MXene features in the AFM topography of m-MXene (Figure 11i). This dual treatment suppressed both lateral and vertical moisture diffusion while increasing the work function from 4.89 eV for h-MXene to 5.84 eV for m-MXene, with a sheet resistance of 97.4 Ω/sq.

The high work function of m-MXene facilitated hole injection into the Super Yellow emissive layer, resulting in higher current density and improved current efficiency compared with ITO- and h-MXene-based OLEDs (Figure 11j). Environmental stability measurements further showed that the sheet resistance of m-MXene increased by only 4.8% and its work function remained above 5.60 eV after 22 days under ambient conditions, whereas untreated and solely annealed MXene electrodes exhibited greater electrical degradation or lower work functions (Figure 11k). The electrode was subsequently integrated into flexible OLEDs on 6 × 6 cm^2^ PET substrates, including a 10 × 10 passive-matrix array that maintained spatially uniform emission under both inward and outward bending (Figure 11l) [170].

Overall, these studies demonstrate that MXene-based electrodes can be engineered through hybridization, polymer passivation, and surface energy control to meet the electrical, optical, mechanical, and environmental requirements of flexible light-emitting devices [177,187]. Such integrated material strategies provide a practical route toward scalable, solution-processed OLEDs and wearable display platforms beyond brittle ITO-based architectures [3,188].

## 5. Synergistic Multifunctional Integrated Systems

### 5.1. Self-Powered Systems

Self-powered optoelectronic systems integrate ambient energy harvesting with signal detection, providing an attractive route toward autonomous wearable and distributed sensing platforms [189,190]. Beyond serving as conductive electrodes, MXenes can actively regulate interfacial polarization [189,191], carrier-transport barriers [192,193], and electromechanical coupling through their termination-rich surfaces [33,35]. These properties enable the integration of piezoelectric [194], pyroelectric [190], triboelectric [26,34,195], and photoelectric processes within compact device architectures [27,32,196]. Recent MXene-enabled self-powered systems, their operating mechanisms, and representative performance characteristics are summarized in Table 9.

**Figure 12:**
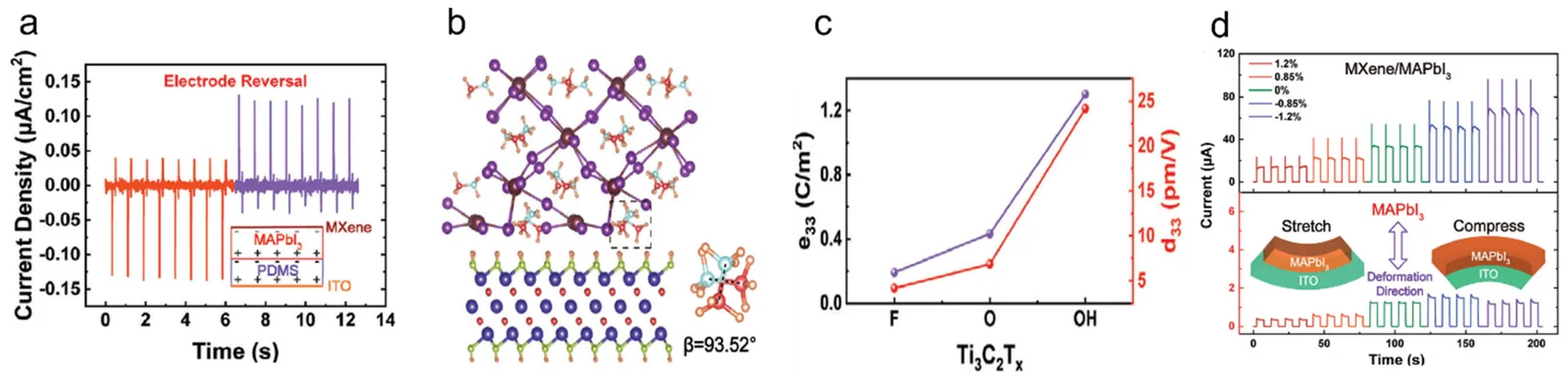
(**a**) Output current characteristics of PENG. (**b**) Polarization alignment at the MXene–perovskite interface induced by hydroxyl group (−OH) interactions with MA^+^ molecules. (**c**) Piezoelectric coefficient (*d*_33_) as a function of MXene surface functional groups. (**d**) Photocurrent of the photodetector with and without the MXene layer under different strain conditions. Reproduced with permission from Ref. [198]. Copyright (2024) Wiley-VCH GmbH.

Han et al. introduced a Ti_3_C_2_T_x_ interlayer between ITO and MAPbI_3_ to enhance the piezoelectric response of the perovskite and subsequently applied the heterostructure to a flexible self-powered photodetector (Figure 12a–d) [198]. The MXene/MAPbI_3_ film exhibited a piezoelectric coefficient of 18.28 pm V^−1^, compared with 5.66 pm V^−1^ for pristine MAPbI_3_. A piezoelectric nanogenerator based on an ITO/MXene/MAPbI_3_/PDMS/ITO structure consequently generated outputs of up to 0.203 μA cm^−2^ and 3.71 V, while electrode-reversal measurements confirmed the piezoelectric origin of the signals (Figure 12a).

DFT calculations revealed that this enhancement was strongly governed by the surface terminations of MXene (Figure 12b,c). F-rich surfaces rotated the MA^+^ dipoles against the spontaneous polarization of MAPbI_3_ and reduced the *d*_33_ value to 4.16 pm V^−1^, whereas O-rich surfaces produced only a moderate increase to 6.86 pm V^−1^. In contrast, OH-rich MXene promoted favorable dipole–dipole interactions and more ordered MA^+^ alignment, increasing *d*_33_ to 24.15 pm V^−1^, approximately 4.27 times that of pristine MAPbI_3_. However, because excessive OH groups induced nanosheet aggregation and deteriorated film uniformity, conventional mixed-termination Ti_3_C_2_T_x_ was selected for device integration.

In the resulting self-powered photodetector, the MXene interlayer facilitated electron transport while strain-induced polarization charges modulated the back-to-back Schottky barriers at the MXene/MAPbI_3_ and MAPbI_3_/Au interfaces. Under 405 nm illumination at zero bias, compressive strain progressively increased the photocurrent, with −1.2% strain producing a 102% enhancement in responsivity relative to the unstrained state (Figure 12d). The combined piezo-phototronic and pyro-phototronic effects also reduced the response time to approximately 2 ms, demonstrating how termination-controlled MXene interfaces can couple mechanical energy harvesting with high-speed optical detection.

In another representative study, Ghosh et al. developed a stretchable triboelectric nanogenerator based on a hierarchically porous TPU composite containing ferroelectric BaTiO_3_ nanoparticles and Ti_3_C_2_T_x_ nanosheets. MXene increased the dielectric permittivity through microcapacitor formation, while BaTiO_3_ polarization suppressed leakage and stabilized interfacial charges, reducing dielectric loss from 0.77 to 0.24 while maintaining a dielectric constant of approximately 30. Combined with a 65% porous structure and interlocked microdome architecture, this tribo-ferroelectric coupling increased the transferred charge density to 2.17 mC m^−2^, produced a low-pressure sensitivity of 4.6 V kPa^−1^, and achieved an energy-conversion efficiency of approximately 79%. The large-area device generated an open-circuit voltage of approximately 260 V and a maximum power density of 6.65 W m^−2^, while remaining operational under strains of up to 60%. Its ability to resolve wrist-pulse waveforms before and after exercise further demonstrated the potential of MXene–ferroelectric composites for self-powered, stretchable physiological sensing [206].

Taken together, these studies show that MXene can serve not only as a conductive component but also as an active regulator of interfacial polarization [189], dielectric loss [209], and charge transport in self-powered devices [210,211]. The coordinated design of MXene interfaces [14,192], ferroelectric components [26,190,194], and deformable microstructures offers a practical route toward autonomous wearable sensors [34,195], human–machine interfaces [33,36], and distributed electronics operating without external power supplies [190,196].

### 5.2. Neuromorphic Perception Systems

Neuromorphic perception systems integrate optical sensing, information processing, and memory within a unified device architecture, thereby reducing the data-transfer and energy costs associated with conventional sensing and computing hardware [28,37]. MXenes are well suited to these systems because their conductivity, surface chemistry, oxidation state, and heterointerfacial electronic structure can be engineered to regulate photocarrier generation, trapping, and relaxation [38,41,193]. These characteristics enable light-induced conductance states to be retained and reinforced, reproducing key functions such as perception, learning, forgetting, and memory consolidation [39,212]. This subsection highlights representative MXene-based optoelectronic synapses and perception systems, while their operating mechanisms and computing performances are summarized in Table 10.

Sha et al. developed a flexible ultraviolet optoelectronic synapse based on a CuSCN/MXene–TiO_x_/TiO_2_ heterostructure, in which partially oxidized MXene acted as both an electron-transport pathway and a defect-rich charge-storage layer. Oxygen vacancies and hydroxyl-related states in the MXene–TiO_x_ structure captured photogenerated carriers and released them gradually after illumination, producing persistent photoconductivity and transitions from short- to long-term memory. The device exhibited paired-pulse facilitation, 30 distinguishable conductance levels corresponding to approximately 5-bit weight resolution, efficient relearning, and stable postsynaptic responses after 1000 bending cycles. Its experimentally measured potentiation and depression characteristics were further implemented in a multilayer neural network, yielding recognition accuracies of 94.60% for MNIST and 79.81% for Fashion-MNIST, with a minimum pulse energy of 6.508 pJ. These results demonstrate that controlled MXene oxidation can integrate photocarrier transport, defect-mediated memory, flexible sensing, and neuromorphic computation within a single optoelectronic platform [220].

**Figure 13:**
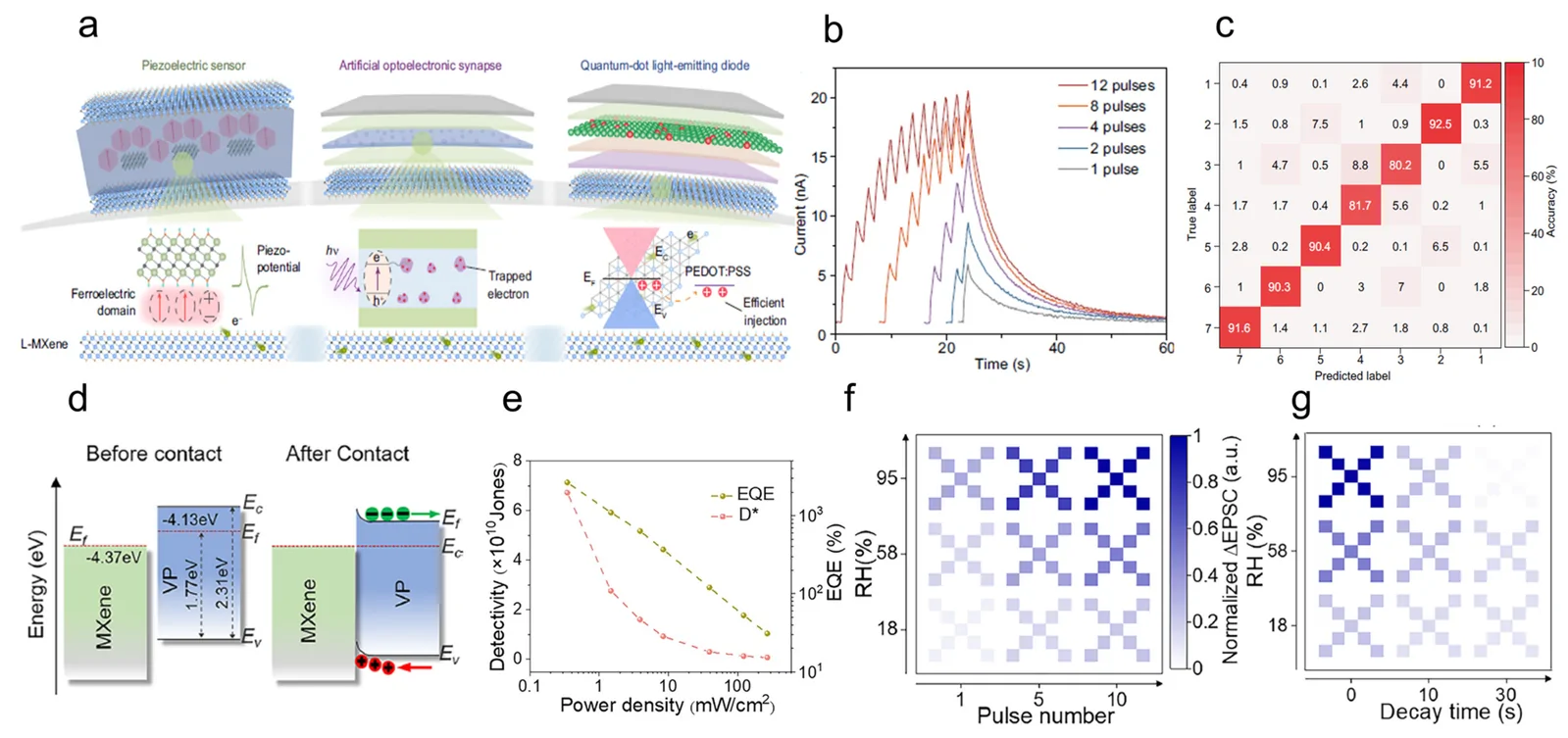
(**a**) Schematic of the MXene-based flexible sensing-processing-visualizing integrated system. (**b**) Cumulative EPSC response as a function of light pulse number. (**c**) Accuracy of multimodal image recognition using tactile and visual dual-mode inputs. Reproduced with permission from Ref. [221]. Copyright (2026) Springer Nature. (**d**) Energy band diagram of MXene/VP heterojunction before and after contact. (**e**) Photoresponsivity characteristics of the MXene/VP device. (**f**) Visual pattern recognition under varying humidity conditions. (**g**) Humidity-dependent forgetting process of visual memory. Reproduced with permission from Ref. [212]. Copyright (2024) Springer Nature.

Wang et al. developed a bioinspired flexible sensing–processing–visualizing system in which size- and surface-state-engineered Ti_3_C_2_T_x_ MXenes performed distinct functions within a mechanically and electrically compatible platform (Figure 13a). Large-sized MXene served as a shared transparent electrode and conductive interconnect, exhibiting 80.4% transmittance and a sheet resistance of approximately 64 Ω sq^−1^. Medium-sized MXene enriched with −OH terminations promoted interfacial polarization locking in P(VDF-TrFE), enhancing the piezoelectric response of the tactile-sensing unit. In parallel, oxidized small-sized MXene provided photoresponsive defect states for charge trapping in the artificial optoelectronic synapse, while the color-shifting quantum dot light-emitting diode converted the processed electrical signals into visible optical feedback. This MXene-centered architecture therefore integrated tactile sensing, visual information processing, and visualization without relying on mechanically mismatched electrode materials.

The artificial optoelectronic synapse exhibited a pulse number-dependent photoresponse under 365 nm illumination (Figure 13b). As the number of light pulses increased from 1 to 12, the excitatory postsynaptic current progressively accumulated because photogenerated carriers trapped at the ZnO nanocrystal/oxidized MXene interface were not fully released between successive stimuli. This defect-mediated charge accumulation enabled optical-history-dependent conductance modulation, with a single-pulse energy consumption of 1.094 pJ. When integrated with the piezoelectric nanogenerator, the synaptic unit could also be modulated directly by mechanically generated electrical pulses, enabling self-powered tactile processing and ultra-low-energy optical operation.

The complementary tactile and visual responses were further applied to multimodal image recognition. Partially obscured Fashion-MNIST images were converted into mixed electrical–optical pulse sequences, in which tactile inputs represented the concealed regions and optical inputs encoded the visible regions. Processing these signals through the MXene-based synaptic reservoir yielded a classification accuracy of approximately 91%, as reflected in the confusion matrix in Figure 13c. These findings demonstrate that tailoring MXene size, oxidation state, and surface chemistry can distribute sensing, memory, signal transport, and visual feedback functions across a unified material platform, providing an integrated route toward flexible neuromorphic perception and wearable human–machine interfaces [221].

Ma et al. [212] developed an optoelectronic synapse based on a solution-processed Ti_3_C_2_T_x_ MXene/violet phosphorus van der Waals heterojunction network for visual–olfactory crossmodal perception (Figure 13d–g). The work functions of VP and MXene were determined to be 4.13 and 4.37 eV, respectively. Upon contact, Fermi-level equilibration induced upward band bending in VP and established a built-in electric field directed from VP toward MXene. Under illumination, this interfacial field promoted hole transfer from VP to conductive MXene while retaining electrons in VP, thereby suppressing carrier recombination and facilitating charge transport (Figure 13e).

Owing to this interfacial carrier regulation, the heterojunction exhibited a maximum responsivity of 7.7 A W^−1^ under 360 nm illumination, approximately seven orders of magnitude higher than that of a pristine VP device, together with a detectivity of 6.73 × 10^10^ Jones and an external quantum efficiency of 2.67 × 10^3^% (Figure 13e). The sublinear photocurrent dependence on optical power reflected the combined effects of carrier generation, transfer, trapping, and recombination. Moreover, the slow photocurrent decay, characterized by relaxation times of 1.7 and 24.7 s, provided the persistent photoconductivity required for excitatory postsynaptic current, paired-pulse facilitation, short- and long-term plasticity, and relearning behavior.

The synaptic response was further regulated by relative humidity, which was employed as a model olfactory input. Increasing humidity reduced the dark current and enhanced the normalized postsynaptic response during repeated optical stimulation, producing stronger recognition of an X-shaped visual pattern (Figure 13f). However, water-related ionic species accelerated the disappearance of trapped photocarriers, resulting in faster memory decay at higher humidity (Figure 13g). Thus, the same visual stimulus was perceived more strongly but forgotten more rapidly as the simulated olfactory-input level increased, demonstrating proof-of-concept crossmodal modulation within a two-terminal optoelectronic synapse [212].

More broadly, MXene-based neuromorphic perception systems illustrate how interfacial carrier transport, defect-mediated memory, and environmental responsiveness can be integrated within compact optoelectronic architectures [28]. Such convergence provides a materials-level pathway toward multimodal artificial perception [37,222], adaptive sensory processing [38], and energy-efficient human–machine interfaces [195,222].

## 6. Summary and Perspective

Interfacial chemistry has served throughout this review as the framework connecting the fundamental properties of MXenes to their behavior in multifunctional optoelectronic systems. Metallic charge transport, a composition-dependent optical response, and a chemically programmable surface give MXenes an uncommon combination of conductivity, optical activity, mechanical compliance, and solution processability. The more decisive point is that surface terminations and interfacial dipoles allow the work function, energy-level alignment, Schottky barriers, and carrier-transfer kinetics to be tailored for a given heterojunction. Molecular functionalization, noncovalent doping, termination regulation, defect passivation, and hybrid interface construction have therefore turned MXenes from passive conductive additives into active interfacial materials that govern charge injection, extraction, recombination, ion migration, and environmental degradation. On this basis, they have been integrated as transparent electrodes, transport layers, photothermal components, charge-trapping media, and multifunctional interfaces in solar cells, photodetectors, light-emitting devices, transparent thermal platforms, self-powered systems, and neuromorphic perception architectures. What these advances show is that the technological value of MXenes lies not in replacing conventional conductors, but in coordinating photons, electrons, ions, heat, and mechanical stimuli through chemically engineered interfaces.

Further development of MXene optoelectronics will depend on deterministic control of the surface composition and of the interfacial evolution that occurs under realistic operating conditions. Ambient oxidation, stochastic mixed terminations, batch-dependent work functions, interflake restacking, and limited large-area uniformity remain the main barriers to reproducible device integration [223,224]. Priorities should therefore include fluorine-free and termination-selective synthesis, passivation layers that resist oxidation while remaining electronically accessible, scalable ink formulations, and in situ and time-resolved characterization under combined illumination, electrical bias, temperature, strain, and humidity [224]. Opportunities also extend beyond the established device categories. The broadband optical response, photothermal conversion, tunable dielectric function, and stimulus-responsive surface chemistry of MXenes suit reconfigurable photonics, multilevel optical encryption, dynamic anti-counterfeiting, and physically unclonable security platforms [225,226]. Moisture energy harvesting deserves particular attention, since hydrogen bonding, water adsorption, interlayer ion transport, and asymmetric MXene interfaces may be used to convert humidity gradients into electrical energy instead of being treated solely as degradation pathways [223,227,228]. Combining these emerging functions with low-power photodetectors, displays, synaptic devices, and energy-storage components could yield closed-loop platforms that harvest ambient energy, perceive multimodal information, perform local computation, and generate adaptive visual feedback [223,224,225]. Progress of this kind would establish MXenes as a systems-level materials platform for scalable, autonomous, and intelligent optoelectronics rather than as components confined to individual devices.

## Figures and Tables

**Table 1:** Structural and optical properties of MXenes.

Materials	Process Method	Structure	Performance	Characteristics	Ref.
Ti_3_C_2_T_x_	Etching: HF+HCl	Spin-coated film Thickness: 19 nm	Extinction Peak Wavelength: ≈760 nm → ≈800 nm (0–400 °C) Extinction amplitude: - max ≈ 2.2 (100 °C) - min ≈ 1.0 (400 °C)	As temperature increases, oxidation reduces free carrier density, leading to a decrease in the extinction coefficient.	[69]
	Etching: LiF+HCl spontaneous delamination: centrifugation and DI water cleaning and sonication	Thickness = 56 ± 2 nm	Static absorbance peak ≈ 750 nm (1.65 eV)	Optical excitation induces absorption bleaching centered at the weak absorption peak observed in the static spectrum, indicating a localized surface plasmon resonance (LSPR). Low thermal conductivity results in slow lattice cooling.	[66]
	LiF+HCl etching → Delamination → Vacuum filtration	Layer thickness: ~1 nm film thickness: ~15 µm 2*θ* = 6.5° ((002) plane peak)	Absorptance ≈ 90% Top *ε* ≈ 17 ± 2% Bottom *ε* ≈ 10 ± 1% *η* ≈ 78%, ΔT ≈ 62 °C	Low IR emissivity comparable to metals. The absorption and emission properties of MXenes depend on the orientation of nanoflakes and surface terminations.	[117]
	Etching: HF Delamination: TMAOH + sonication	Spray-coated film	The onset of the decrease in transmittance: 1250 nm (0.99 eV) Peak transmittance ≈ 60% (at ≈1300 nm) Peak reflectance: 720 nm Extinction peak: *λ*_max_ = 800 nm	Color of MXene colloids and films: forest green/dark purple. The transmittance decreases with wavelength (NIR). As the thin film thickness increases, the visible transparency (at 550 nm) decreases.	[51]
	Etching: HF, HCl Delamination: LiCl	(002) diffraction peak in the 2*θ* range of 4.8–7.6°	The average infrared emissivity (3–25 μm): 0.06	Emissivity shows an inverse dependence on electrical conductivity. Overall, very low emissivity, indicating strong metallic character.	[55]
	Etching: LiF/HCl Self-assembly on the substrate	PDDA → MXene → PDDA → MXene … (N = 5–30)	N ↑ (5 → 30), optical bandgap ~1 eV → ~0.66 eV Transmittance: >60% (400–1800 nm, 5-layer sample) K (≈800 nm): ≈0.49, ≈0.53 (5 and 30 layers respectively) Nonlinear absorption coefficient *β* (≈800 nm, ≈140 fs, ≈80 MHz): 5 layers ≈ 7.13 × 10^2^ cm/GW 30 layers ≈ −2.69 × 10^2^ cm/GW	The linear absorption increased with the increase in layer number N. The transmittance decreased with an increasing layer number. The k value slightly increases with increasing layer number. As the layer number increases from 5 to 30, the film transitions from RSA to SA.	[70]
	Etching: LiF/HCl Delamination: LiCl Spin-coating on a quartz substrate	Thin Film: 5–30 nm Bulk Film: 450–950 nm =O: -OH: -F = 18.1: 16.9: 21.0 Average size: 6.6 ± 1.5 μm	Lorentz oscillator parameters NIR-UV Thin Film: A = 30 at E = 0.312 eV Bulk Film: A = 12.15 at E = 0.417 eV Mid-IR Thin Film: A = 70 at E = 0.165 eV Bulk Film: A = 64 at E = 0.10 eV	Visible–NIR → plasmonic modes. Mid-IR → band-to-band transition-like resonance. Increasing thickness enhances both *ε*_1_ (metallicity) and *ε*_2_ (absorption/loss).	[85]
	Substrate fabrication: photolithography + dry etching (3D trench formation) MXene: - Al etching - Deposition: Spin coating (controlled coverage)	3D Trench Structure Size ≈ 1 μm Thickness ≈ 1.6 nm	Flat structure → Trench structure - Surface EF peak (at coverage 50%) ≈ 2.5 × 10^2^ → 10^2^ - Edge EF peak ≈ 3 × 10^4^ → 9 × 10^4^	Sub-monolayer MXene → edge plasmon generation. Edge plasmon → local field enhancement. Edge plasmon → dominant SERS mechanism. Trench structure → stronger edge hotspots. Optimal coverage (40–60%) → maximum edge effect. Overcoverage → reduced edge length, weaker SERS.	[56]
	Etching: LiF+H_2_O+HCl liquid/liquid interfacial self-assembly (LLIA)	TiO_2_/Ti_3_C_2_T_x_/PET	Areal Concentration 1 µg/cm^2^: Transmittance (at 550 nm) 96.8% Areal Concentration 60 µg/cm^2^ Transmittance (at 550 nm): 64.3%	Uniform thickness distribution and high single-/double-layer coverage lead to high optical transparency and electrical conductivity.	[43]
	Etching: HCl+LiF Sonication and centrifugation and freezing Spin-coated on PDMS	Isotropic and uniform wrinkled structure with ~50 nm height	Transmittance: 71% Average THz absorption: 42.0% THz Reflection: 83.0% SE_T_: 5.6 dB in Vis range (T = 8 nm)	At lower thickness, the wrinkled structure reduces transmittance and enhances EMI shielding effectiveness. Wrinkled structures improve impedance matching and enhance localized SPP, leading to increased electromagnetic wave absorption.	[49]
Ti_2_CT_x_	Etching: HF+HCl	Spin-coated film Thickness: 3.0 ± 0.2 nm	Extinction Peak Wavelength: ≈540 nm → ≈490 nm (0 –400 °C) Extinction amplitude: - max: ≈5.4 (≈100 °C) - min: ≈2.2 (≈330 °C)	As temperature increases, oxidation reduces free carrier density, leading to a decrease in the extinction coefficient.	[69]
	Etching: HF Delamination: TMAOH + sonication	Spray-coated film	The onset of the decrease in transmittance: 850 nm (1.46 eV) Peak transmittance ≈ 45% (at ≈800 nm) Peak Reflectance spectrum: 442 nm Extinction peak: λ_max_ = 542 nm	Color of MXene colloids and films: dark purple/green. The transmittance decreases with wavelength (NIR). As the thin film thickness increases, the visible transparency (at 550 nm) decreases.	[51]
	Etching: HF, HCl Delamination: LiCl	D-spacing: 11.62 Å (002) diffraction peak in the 2*θ* range of 4.8–7.6°	The average infrared emissivity (3–25 μm) < 0.5	Thermal radiation can be suppressed during radiative heating. Thermal radiation can be suppressed during radiative heating at a wavelength of 25 μm.	[55]
Ti_3_CNT_x_	Etching: HF Delamination: TMAOH	Nanosheet Average Thickness: 2.6 nm	At 800 nm *β* = −0.054 cm/GW(SA) At 1550 nm *β* = 0.31 cm/GW(RSA)	Ground-state bleaching (GSB) is attributed to a dynamic Burstein–Moss effect caused by photoexcited carrier filling and Pauli blocking. SA is observed under 500 and 800 nm and RSA is observed under 1300 and 1550 nm laser irradiation.	[118]
	Etching: HF, HCl Delamination: LiCl	(002) diffraction peak in the 2*θ* range of 4.8–7.6°	The average infrared emissivity (3–25 μm) < 0.5	Thermal radiation can be suppressed during radiative heating at a wavelength of 25 μm. Emissivity shows an inverse dependence on electrical conductivity.	[55]
Nb_2_CT_x_	Etching: HF sonication and centrifugation, DI water cleaning Dispersion TMAOH	Thickness = 164 ± 20 nm	Static absorbance peak ≈ 730 nm (1.7 eV) Band gap = 60 meV (N-terminated)	Optical excitation induces absorption bleaching centered at the weak absorption peak observed in the static spectrum, indicating a localized surface plasmon resonance (LSPR).	[66]
	Etching: HF Delamination: TMAOH	Spray-coated film-	Extinction peak ≈ 915 nm Peak transmittance ≈ 65% (at ≈2000–2500 nm)	Color of MXene colloids and films: blue/golden yellow. The transmittance increases with wavelength (NIR). As the thin film thickness increases, the visible transparency (at 550 nm) decreases.	[51]
	Etching: HF Delamination: TMAOH	(002) diffraction peak in the 2*θ* range of 4.8–7.6°	The average infrared emissivity (3–25 μm): 0.59	Emissivity shows an inverse dependence on electrical conductivity.	[55]
Nb_4_C_3_T_x_	Etching: HF Delamination: TMAOH	Spray-coated film	No distinct extinction peak was observed	Color of MXene colloids and films: grey–brown/grey–black.	[51]
	Etching: HF Delamination: TMAOH	(002) diffraction peak in the 2*θ* range of 4.8–7.6°	The average infrared emissivity (3–25 μm) > 0.5	Emissivity shows an inverse dependence on electrical conductivity.	[55]
V_2_CT_x_	Etching: HF Delamination: TMAOH	Spray-coated film	The onset of the decrease in transmittance: 600 nm (2.07 eV) Peak transmittance ≈ 40% (at ≈400 nm) No distinct extinction peak was observed	Color of MXene colloids and films: green–blue/bronze. The transmittance decreases with wavelength (NIR). As the thin film thickness increases, the visible transparency (at 550 nm) decreases.	[51]
	Etching: HF Delamination: TMAOH	D-spacing: 12.98 Å (002) diffraction peak in the 2*θ* range of 4.8–7.6°	The average infrared emissivity (3–25 μm) < 0.5	Thermal radiation can be suppressed during radiative heating. Thermal radiation can be suppressed during radiative heating at a wavelength of 25 μm. strong metallic response in the near-infrared (NIR) region.	[55]
V_4_C_3_T_x_	Etching: HF Delamination: LiCl	(002) diffraction peak in the 2*θ* range of 4.8–7.6°	The average infrared emissivity (3–25 μm) < 0.5	Thermal radiation can be suppressed during radiative heating. Thermal radiation can be suppressed during radiative heating at a wavelength of 25 μm.	[55]
Mo_2_CT_x_	Etching: HF Delamination: TMAOH	Spray-coated film	Extinction peak ≈ 450 nm Peak transmittance ≈ 90% (at ≈2000 nm)	Color of MXene colloids and films: brown/silver. The transmittance increases with wavelength (NIR). As the thin film thickness increases, the visible transparency (at 550 nm) decreases.	[51]
Mo_2_TiC_2_T_x_	Etching: HF Delamination: TMAOH	Spin-coated film Thickness: 9.0 ±1.9 nm	Extinction Peak Wavelength: ≈480 nm → ≈520 nm (0 –400 °C) Extinction amplitude: - max ≈ 3.9 (100 °C) - min ≈ 1.2 (400 °C)	As temperature increases, oxidation reduces free carrier density, leading to a decrease in the extinction coefficient.	[69]
	Etching: HF Delamination: TMAOH	Spray-coated film	Extinction peak ≈ 476 nm Peak transmittance ≈ 75% (at ≈2250 nm)	Color of MXene colloids and films: orange–brown/light blue–silver. The transmittance increases with wavelength (NIR). As the thin film thickness increases, the visible transparency (at 550 nm) decreases.	[15]
Mo_2_Ti_2_C_3_T_x_	Etching: HF sonication and centrifugation, DI water cleaning	Thickness = 64 ± 10 nm	Static absorbance peak ≈ 530 nm (2.38 eV)	Optical excitation induces absorption bleaching centered at the weak absorption peak observed in the static spectrum, indicating a localized surface plasmon resonance (LSPR). Low thermal conductivity results in slow lattice cooling.	[66]
	Etching: HF Delamination: LiCl	(002) diffraction peak in the 2*θ* range of 4.8–7.6°	The average infrared emissivity (3–25 μm) < 0.5	Emissivity shows an inverse dependence on electrical conductivity. Thermal radiation can be suppressed during radiative heating at a wavelength of 25 μm.	[55]
Ta_4_C_3_T_x_	Etching: HF Delamination: TMAOH	Spray-coated film	No distinct extinction peak was observed	Color of MXene colloids and films: brown/silver–gray Ta_4_C_3_ shows low extinction, implying low optical losses for photonic applications such as transparent conductors.	[51]
Ti_2.5_Ta_2.5_C_4_	Etching: HF Delamination: TMAOH	(002) peak: 6.10° *c* lattice parameter: 28.85 Å Flake size: 300 nm	At 550 nm, the extinction coefficient: 18.7 L/g cm Emissivity: ~90% → ~40% (0 μm → 25 μm) Reflectance: ~10% → ~50% (0 μm → 25 μm)	Visually the MXene solutions were black. The infrared emissivity is significantly higher, while the reflectance is significantly lower than that of Ti_3_C_2_.	[114]
Ti_2.675_Nb_2.325_C_4_	Etching: HF Delamination: TMAOH	Average flake thickness: 3.4 nm (002) peak: 5.98° *c* lattice parameter: 28.88 Å Average flake size > 500 nm	At 550 nm, the extinction coefficient: 20.9 L/g cm Reflectance: ~10% → ~50% (0 μm → 25 μm)	Visually the MXene solutions were black The infrared emissivity is significantly higher, while the reflectance is significantly lower than that of Ti_3_C_2_.	[114]
Ti_0.4_Nb_1.6_CT_x_	Etching: HF Delamination: TMAOH	(002) diffraction peak in the 2*θ* range of 4.8–7.6°	The average infrared emissivity (3–25 μm) > 0.5	Emissivity shows an inverse dependence on electrical conductivity. The IR emissivity increases with increasing Nb content.	[55]
Ti_1.6_Nb_0.4_CT_x_	Etching: HF, HCl Delamination: LiCl	(002) diffraction peak in the 2*θ* range of 4.8–7.6°	The average infrared emissivity (3–25 μm) < 0.5	Thermal radiation can be suppressed during radiative heating at a wavelength of 25 μm. Emissivity shows an inverse dependence on electrical conductivity. The IR emissivity increases with increasing Nb content.	[55]

**Table 2:** Conductivity-dependent properties of MXenes.

Materials	Process Method	Type	Performance	Characteristics	Ref.
Ti_3_C_2_T_x_	Etching: HF + HCl	Spin-coated film Thickness: 19 nm	Optical resistivity: 0.0010 ± 0.0001 Ohm cm	As the MXene film becomes thinner, the resistivity of the material decreases. At high temperatures, the resistivity increases sharply as a result of Ti oxidation.	[69]
	Etching: HF Delamination: TMAOH	Spray-coated film	Carrier concentration ≈ 3 × 10^22^ cm^−3^ (HF/TMAOH) DC conductivity = 411 ± 49 S/cm (HF/HCl/LiCl) DC conductivity = 7310 ± 120 S/cm	The etching and processing conditions directly affect the charge carrier transport properties of the produced flakes.	[51]
	Etching: LiF/HCl Delamination: LiCl Spin-coating on a quartz substrate	Thickness - Thin Film: 5–30 nm - Bulk Film: 450–950 nm	Resistivity: Thin Film: 2.91 × 10^−5^ Ω.cm Bulk Film: 29.37 × 10^−5^ Ω.cm Scattering Time (fs) Thin Film: 178.49 Bulk Film: 51.36	A transition from dielectric to metallic behavior occurs at ~1.01 μm wavelength for thin films and ~1.35 μm wavelength for bulk-like films.	[85]
	Al-rich MAX phase synthesis Etching: HCl + H_2_O + HF (6:3:1) Delamination: LiCl	Free standing film Average flake size: 1.3–1.6 μm Max size > 25 μm	*σ* > 20,000 S/cm(initial) *σ* ≈ 10,000 S/cm (films from 4-month-stored solution)	Oxidation is delayed at elevated temperatures due to reduced defects and improved Ti:C stoichiometry. Storage stability exceeding 10 months.	[63]
	Etching: LiF + H_2_O + HCl liquid/liquid interfacial self-assembly (LLIA)	2D thin film Thickness ≈ 2 nm	Transmittance: Sheet resistance (*R*_s_) 96.9%: 1623 Ω/sq 94.1%: 210 Ω/sq 87.8%: 123 Ω/sq	Uniform thickness and high single-/double-layer coverage → high transparency and conductivity. Lower sheet resistance than other fabrication methods and transparent conductive materials.	[43]
	Etching: HCl + LiF Sonication and centrifugation and freezing Spin-coated on PDMS	Wrinkle Film Thickness: ~8 nm (intrinsic thickness of film) Wrinkle height: ~50 nm (surface corrugation amplitude)	Sheet resistivity ≈ 200 Ω/sq Charge carrier scattering time: 8.0 fs Charge carrier density: 9.4 × 10^27^ Surface electron density: 7.5 × 10^19^ m^−2^	Reflectance and transmittance show negative and positive correlations with sheet resistance, respectively. A wrinkled structure can enhance the electrical conductivity of MXene films, thereby improving impedance matching.	[49]
	Etching: HF + HCl Delamination: LiCl solution in DI water and centrifugation. Ti_3_C_2_T_x_ film: interfacial self-assembly on Si substrate	Self-assembled film Monolayer thickness: 2.5 nm	*σ*_DC_ ≈ 2.5 ms–30 ms (1–25 layers) *τ* ≈ 13.9–19 (1–25 layers) Mobility ≈ 85–115 cm^2^/Vs	The real conductivity increases with increasing layer numbers, while the imaginary conductivity decreases. In-plane conductivity exceeds out-of-plane conductivity. As the substrate dielectric constant increases, charge carrier scattering increases, leading to a decrease in electrical conductivity.	[64]
	Etching: MILD method Delamination: H_2_O cleaning, centrifugation. Si/SiO_2_ deposition: drop casting	Thin Films and Networks *c* lattice parameter: 25.2 Å	Contact resistivity: 2.5 Ω Voltage drop = 1.8 eV (at the inter-flake junction) Resistivity drop = 18.4 kΩ (at the inter-flake junction)	Resistance increase occurs by steps corresponding to inter-flake junctions. High junction resistance is attributed to out-of-plane resistivity anisotropy and constriction resistance governed by the effective electrical contact area at flake interfaces.	[124]
	Etching: HF, HCl, DI water	Spray-coated film Thickness: 25 nm	Mean free path: ~14 nm (at 300K), ~23 nm (100k) At room temperature - Mobility: ~70 cm^2^/V·s - Carrier density: ~1.2 × 10^21^ cm^−3^ - static conductivity ≈ 1.75 × 10^6^ S/m At 300k - static conductivity ≈ 1.25 × 10^6^ S/m	Scattering time decreases with increasing temperature, indicating band-like charge transport. Reduced phonon population at lower temperatures suppresses scattering, enhancing carrier mobility and conductivity. Carrier density remains nearly constant; conductivity is governed by scattering time.	[130]
	Etching: HF Centrifugation and vacuum filtration	Paraffin Composite	*ε*′ (real permittivity) = 3.8–4.0 *ε*″ (dielectric loss) = 0.30–0.60 *µ*′ (real permeability) = 0.98–1.08 *µ*″ (magnetic loss) < 0.2 Minimum RL: −38.7 dB (1.7 mm thickness)	Impedance mismatch. Energy dissipation mainly relies on conductive loss.	[129]
	Etching: LiF + HCl Centrifugation and DI water cleaning Spin coating on quartz substrate	Spin-cast Thin Film	Transmittance 72% → ~94% ⇒ *R*_s_: 2010 → 23,660 Ω/sq Transmittance 89.9% → 99.8% ⇒ *R*_s_ 11,870 → 23,660 Ω/sq FoM: 2.027 ± 0.163	Spin speed increased from 2000 to 4000 rpm, resulting in decreased film thickness, increased transparency and increased sheet resistance.	[50]
Ti_2_CT_x_	Etching: HF/HCl/LiCl or HF/TMAOH	Ultrathin film	Optical resistivity: 0.00015 ± 0.00001 Ohm cm (Thickness: 3 nm)	As the MXene film becomes thinner, the resistivity of the material decreases. At high temperatures, the resistivity increases sharply because of Ti oxidation.	[69]
Ti_2_NbC_2_T_x_	Etching: HF Centrifugation and vacuum filtration	Paraffin Composite	*ε*′ (real permittivity) = 4.5 *ε*″ (dielectric loss) = 0.13–0.25 *µ*′ (real permeability = 0.98–1.08 *µ*″ (magnetic loss) < 0.2	Suppression of Ti oxidation *via* electron cloud modulation by Nb doping. It exhibits enhanced interfacial polarization and a relatively high dielectric constant. Impedance mismatch.	[129]
Ti_2_TaC_2_T_x_	Etching: HF Centrifugation and vacuum filtration	Paraffin Composite	*ε*′ (real permittivity) ≈ 3.4 *ε*″ (dielectric loss) = 0.10–0.18 *µ*′ (real permeability = 0.98–1.08 *µ*″ (magnetic loss) < 0.2	Suppression of Ti oxidation *via* electron cloud modulation by Ta doping. loss mechanisms are inefficient and its impedance matching is poor.	[129]
Ti_2_VC_2_T_x_	Etching: HF Centrifugation and vacuum filtration	Paraffin Composite	*ε*′ (real permittivity) = 3.8–3.9 *ε*″ (dielectric loss) = 0.18–0.36 *µ*′ (real permeability = 0.98–1.08 *µ*″ (magnetic loss) < 0.2 Minimum RL: −48.4 dB Frequency range: 9–12 GHz	Absorption arises from both conductive and polarization loss mechanisms. Shows strong orbital hybridization between Ti and V.	[129]
Nb_2_CT_x_	Etching: HF Centrifugation and vacuum filtration	Paraffin Composite	*ε*′ (real permittivity) = 3.4–3.45 *ε*″ (dielectric loss) = 0.11–0.21 *µ*′ (real permeability = 0.98–1.08 *µ*″ (magnetic loss) < 0.2 Minimum RL: −36.4 dB (1.8 mm thickness)	Absorption is limited due to insufficient conductive and polarization losses.	[129]
Nb_4_C_3_T_x_	Etching: LiF Delamination: TMAOH + sonication	Thin-film network	*σ* = 0 (upon photoexcitation) Band gap: 0.19 eV (Tauc plot method)	Semiconducting behavior. Free carrier conductivity (or absorption) is negligible in the 0–2 THz range (0–8 meV). Charge transport is governed by band-like conduction within flakes and hopping-limited transport between flakes.	[125]
V_2_CT_x_	Etching: HF Centrifugation and vacuum filtration	Paraffin Composite	*ε*′ (real permittivity) ≈ 3.0 *ε*″ (dielectric loss) = 0.6 (at high frequency) *µ*′ (real permeability = 0.98–1.08 *µ*″ (magnetic loss) < 0.2 Minimum RL: −53.8 dB (1.5 mm thickness) Frequency range: 8.2–11.5 GHz (broad low-RL region)	Excellent impedance matching (near (1,0)). Microwave absorption is governed by synergistic carrier-mediated and polarization loss mechanisms.	[129]
Mo_2_Ti_2_C_3_T_x_	Etching: HF Delamination: TMAOH	Ultrathin film Thickness: 9 nm	Optical resistivity: 0.0062 ± 0.0013 Ohm cm	As the MXene film becomes thinner, the resistivity of the material decreases. TMA^+^ intercalation increases interlayer spacing, leading to higher electrical and optical resistivity. At high temperatures, the resistivity increases sharply because of Ti oxidation.	[69]

**Table 3:** Surface termination-induced chemical and electronic changes.

Materials	Calculation Method	Structure	Performance	Characteristics	Ref.
Ti_3_C_2_T_x_	DFT within the local density approximation (LDA)	Boundary condition Residual forces: < 10^−2^ eV Å^−1^ Total energy convergence: < 10^−2^ eV.	WF = 3.69 eV (F:OH:O = 62:25:13) Interface dipole *p*^OH/O^ ≈ 6 (OH:O 100:0) *p*^OH/O^ ≈ 0 (OH:O 0:100)	OH termination drives charge transfer from H to O, forming a surface dipole that lowers the vacuum level and hence the WF, whereas O termination produces the opposite surface polarization and raises it. The WF depends nonlinearly on the OH ratio, reaching a minimum near 75% OH owing to interactions between the mixed terminations.	[111]
	UPS Spectrum	D-Ti_3_C_2_T_x_: Ti_3_C_2_T_x_ treated with 50 mg/mL ethanolamine solution R- Ti_3_C_2_T_x_: Ti_3_C_2_T_x_ treated with a 15 mg/mL RhCl_3_ solution	Ti_3_C_2_T_x_ WF ≈ 4.80 eV Ti_3_C_2_T_x_ treated with ethanolamine WF ≈ 4.23 eV Ti_3_C_2_T_x_ treated with RhCl_3_ WF ≈ 5.04 eV	Ethanolamine treatment induces interfacial dipole formation between −NH_2_ groups and the −O/−OH/−F groups on Ti_3_C_2_T_x_, causing a downshift of the vacuum level, while electron donation from −NH_2_ leads to an upshift of the Fermi level. RhCl_3_ treatment causes electron transfer from Ti_3_C_2_T_x_ to Rh^3+^, reducing it to Rh^0^ and decreasing the electron density of Ti_3_C_2_T_x_, which leads to a downshift of the Fermi level.	[98]
	Z-scan + DFT calculations performed using the Quantum ESPRESSO code	Ti_3_C_2_T_x_ (pristine): open circuit voltage Ti_3_C_2_T*_x_* (−OH rich): negative V_A_ (≈−0.8 V) Ti_3_C_2_T*_x_* (=O rich): positive V_A_ (≈0.2 V)	Ti_3_C_2_T*_x_* (pristine): RSA (both 515,800 nm) *Β*_eff_ = 153 ± 12.2 cm GW^−1^ (515 nm) Ti_3_C_2_T*_x_* (−OH rich): RSA (both 515,800 nm) *Β*_eff_ = 153 ± 12.2 cm/GW (515 nm) *Β*_eff_ = 94 → 113 cm/GW (800 nm) Ti_3_C_2_T*_x_* (=O rich): SA (515nm) RSA (800 nm) *Β*_eff_ = −1020 ± 136.2 cm/GW (515 nm) *Β*_eff_ = ~67 ± 1.5 cm/GW (800 nm)	Ti_3_C_2_T_x_ (−OH rich) exhibits enhanced excited-state absorption (ESA), resulting in reverse saturable absorption (RSA) with increasing light intensity. Ti_3_C_2_T_x_ (=O rich) shows saturable absorption (SA) due to Pauli blocking, where state filling suppresses further electronic transitions at high light intensity.	[116]
Sc_2_CT_x_	DFT calculation- HSE06	Halogen-terminated structure	Band gap -Cl 2d Monolayer: 1.69 eV 3D 1-FCC AB: 1.88 eV 3D 2-FCC AB: 1.68 eV -F 2d Monolayer: 1.88 eV 3D 1-FCC AB: 2.38 eV 3D 2-FCC AB: 1.87 eV -Br 2d monolayer: 1.55 eV 3D 1-FCC AB: 1.52 eV 3D 2-FCC AB: 1.39 eV -I 2d monolayer: 0.78 eV 3D 1-FCC AB: 0.64 eV 3D 2-FCC AB: 0.73 eV	A semiconductor characteristic in which both monolayer and multilayer structures exhibit similar band gaps. The band gap decreases as the halogen termination radius increases. Applicable to fields such as photodetectors and photocatalysts.	[133]
Y_2_CT_x_	DFT calculation- HSE06	Halogen-terminated structure	Band gap -F 2d monolayer: 1.91 eV 3D 1-FCC AB: 1.56 eV 3D 2-FCC AB: 1.88 eV -Cl 2d monolayer: 1.69 eV 3D 1-FCC AB: 1.88 eV 3D 2-FCC AB: 1.66 eV -Br 2d monolayer: 1.62 eV 3D 1-FCC AB: 1.67 eV 3D 2-FCC AB: 1.60 eV -I 2d monolayer: 1.23 eV 3D 1-FCC AB: 0.93 eV 3D 2-FCC AB: 1.17 eV	A semiconductor characteristic in which both monolayer and multilayer structures exhibit similar band gaps. The band gap decreases as the halogen termination radius increases. Applicable to fields such as photodetectors and photocatalysts.	[133]

**Table 4:** Interfacial engineering strategies for optoelectronics.

Materials	Structure	Surface Treatment Method	Interfacial Material	Performance	Characteristics	Ref.
	Si/SiO_2_/Ti_3_C_2_T_x_ MXene/PDVT-10/Au	Spontaneous oxidation	Native TiO_2_ (spontaneous oxidation of Ti_3_C_2_T_x_)	*Vth* −1.2 V *J_on_* 5.8 mA/cm^2^ WF 4.6 eV *Φ_B_* 0.39 eV (*V_G_* = −10 V) *Φ_B_* 0.21 eV (*V_G_* = −30 V) *R_q_* 1.81 nm	Ti_3_C_2_T_x_ source electrode with partial screening and mesh-electrode Schottky barrier modulation (SS 73 mV/dec, on/off 2 × 10^5^). Low barrier from the 4.6 eV work function and native TiO_2_ suppresses leakage, giving current saturation and wide-spectrum detection (R 366 A/W, D* 2.8 × 10^12^ Jones).	[134]
	FTO/c-TiO_2/_m-TiO_2/_CsFAMA perovskite/MXene:Me-4PACz/Spiro-OMeTAD/Au	TPAOH/Hydrazine delamination Me-4PACz functionalization *via* bath ultrasonication	MXene:Me-4PACz nanoneedles	PCE 21.47% *V_oc_* 1.16 V *J_sc_* 23.67 mA/cm^2^ FF 78.12% VB −5.6 eV/CB −4.0 eV	Me-4PACz-functionalized MXene at the HTL/perovskite interface, with phosphonic acid intercalation passivating interfacial defects. Improved band alignment (VB: −5.45 → −5.6 eV) and fewer deep and shallow traps, yielding PCE 21.47%, *T*_88_@1000 h (indoor), and *T*_50_@1000 h (outdoor).	[135]
	ITO/PTAA/CsPbI_3_/OMXene-CsPbI_3_ composite/CPTA/BCP/Ag	LiF/HCl etching Heated stirring in NaOH solution for anatase TiO_2_ formation	OMXene-CsPbI_3_ composite layer	PCE 19.69% HOMO −7.39 eV, LUMO −3.90 eV. Single cell (0.096 cm^2^): *V_oc_* 1.21 V, *J_sc_* 19.86 mA/cm^2^, FF 81.96% 25 cm^2^ minimodule: PCE 14.64%, *V_oc_* 3.57 V, FF 71.56% OMXene *E_g_* 3.49 eV	NaOH-oxidized Ti_3_C_2_T_x_ (OMXene) with surface anatase TiO_2_, applied as a composite surface layer on CsPbI_3_ in *p-i-n* PSCs. Work function tuned (−5.37 → −3.91 eV) with a stronger field at the perovskite/ETL interface, giving PCE 19.69%, minimodule 14.64% (25 cm^2^), and 85% retention after 1000 h at 85 °C/85% RH, 1 sun.	[86]
	ITO/MXene doped SnO_2_/Perovskite (CsFAMAPbIBr or FAPbI_3_)/Spiro-OMeTAD/MoO_3_/Ag	LiF/HCl etching Surface functionalization using dodecyltrimethoxysilane to form MXene-H and using FOTS to form MXene-F, respectively	MXene-doped SnO_2_	PCE 23.66% (avg)/24.12% (champion) *V_oc_* 1.118 V (avg)/1.121 V (champion) *J_sc_* 25.44 mA/cm^2^ (avg)/25.49 mA/cm^2^ (champion) WF (SnO_2_-MXene-H) 4.32 eV SnO_2_-M *E_g_* 3.90 eV SnO_2_-MF *E_g_* 3.95 eV SnO_2_-MH *E_g_* 3.92 eV	MXene-H and MXene-F dopants in the SnO_2_ ETL, optimizing surface energy for larger perovskite grains and passivating macroscopic defects. Improved band alignment with a zero Schottky barrier for faster charge transfer, giving champion PCE 24.12% with strong moisture and operational stability.	[145]
	NaHA matrix/MTTTPyB- Ti_3_C_2_ composite	Self-assembly of MTTTPyB molecules onto nanosheets *via* electrostatic interactions	Ti_3_C_2_ MXene/MTTTPyB	Antibacterial rate (*S. aureus*) 97.04% Antibacterial rate (*E. coli*) 83.93% Photothermal conversion efficiency (*η*) 18.29% WF 3.723 → 3.693 eV *E_g_* 1.928 (MTTTPyB) → 1.420 eV (MTTTPyB/Ti_3_C_2_)	MXene-integrated MTTTPyB with an interfacial dipole field that suppresses the excitonic effect and maximizes ROS production. Over 97% antibacterial efficiency from composite-loaded microneedle patches under 660 nm irradiation with photothermal heating.	[95]
	FTO/TiO_2_/NiO/TFA-Ti_3_C_2_T_x_ MXene	MILD etching (LiF/HCl) TFA treatment	Ti_3_C_2_T_x_ MXene treated with trifluoroacetic acid (TFA-MXene)	Photovoltage 0.45 V *J_sc_* 0.2 mA/cm^2^ FF 26.8% PCE (UV) 0.7% *P_max_* 18–30 μW/cm^2^ D* 4.1 × 10^10^ Jones WF ~4.8 eV (cited) Conductivity ~8900 S/cm	TFA-treated Ti_3_C_2_T_x_ transparent electrode (~8900 S·cm^−1^, oxidation-resistant) in an ITO-free transparent PV and self-powered photodetector. WF ~4.8 eV aligned with NiO for hole collection; 39.73% transparency, 80 μs response, 4.1 × 10^10^ Jones.	[6]
	Ti_3_C_2_T_x_ (no device structure; material study only)	HF etching or Molten salt etching Post-annealing under Ar or NH_3_ for N-doping and surface termination control	T_x_ = F, O, N, Cl, or mixed	DFT-calculated WF 4.22–6.31 eV (Cl*- to N*-terminated Ti_3_C_2_) WF tuning range 4.23–4.85 eV	WF tuned over > 0.6 eV by gas-phase annealing (Ar or NH_3_); F* replaced by O* or N* raises the WF, while Cl* lowers it. DFT shows an inverse correlation between termination electron affinity and WF, with TiO_2_ and TiN bulk phases also affecting the final values.	[87]
	MB-doped Ti_3_C_2_T_x_ thin film	molecular doping by immersing films in Magic Blue solution for 2.5–14 h	Magic Blue molecular dopant	WF 4.37 → 4.87 eV (Δ ≈ 500 meV) Resistivity 0.46 → 0.28 mΩ·cm	MB-induced subsurface electron depletion raises the WF by ~120 meV through a Fermi level downshift and interfacial dipoles. Conductivity and plasmonic response are retained, giving termination-independent WF control.	[136]
	Au/MoO_3_-doped Ti_3_C_2_T_x_ MXene/n-Si/In-Ga	LiF/HCl etching Charge-transfer doping by spontaneous adsorption of MoO_3_ nanodots on MXene surface	MoO_3_-doped Ti_3_C_2_T_x_ MXene	WF 4.62 eV → 5.29 eV *R* 110.2 → 494.9 mA/W D* 2.61 × 10^11^ → 1.06 × 10^13^ Jones *ρ* (sheet resistivity) 2.68 × 10^−4^ → 2.04 × 10^−4^ Ω·cm Hole mobility 1.82 → 1.62 cm^2^/V·s	MoO_3_ nanodots spontaneously adsorbed on Ti_3_C_2_T_x_ at 1.0 mg/mL, raising the WF from 4.62 to 5.29 eV by charge-transfer doping. Enlarged built-in potential at the MXene/n-Si interface, giving improved responsivity, detectivity, and LDR with reduced sheet resistivity in a self-driven photodetector.	[94]
	Cr/Au/p-CsCu_2_I_3_/n-Ca_2_Nb_3−x_Ta_x_O_10_/MXene-PEIE (bottom electrode, varied)	LiF/HCl etching 5 nm LiF nanoparticles thermal evaporation 5 nm Se particles thermal evaporation PEIE solution (0.4 wt%) mixed with MXene at 1:1 volume ratio	Ti_3_C_2_T_x_ MXene modified with Se (for high WF) or LiF/PEIE (for low WF)	WF 4.9 → 4.55 eV Rectification ratio 16,136 at ±2 V *Φ_B_* Au 1.37 eV → MXene 1.17 eV → MXene-PEIE 0.82 eV *R* 81.3A/W (250 nm UV) EQE 4.04 × 10^4^%	PEIE coating lowered the MXene WF to 4.55 eV in p-CsCu_2_I_3_/n-Ca_2_Nb_3-x_Ta_x_O_10_ photodetectors, improving hole collection and reducing the Schottky barrier. Rectification ratio 16,136, responsivity 81.3 A/W, with bending stability; applied to UV image sensors and logic gates.	[101]
	MXene/ITO/(p) a-Si:H/(i) a-Si:H/(n) c-Si/(i) a-Si:H/(n) a-Si:H/ITO/MXene	LiF/HCl etching Covalently functionalized with amino acids through self-assembly	Amino acid-functionalized Ti_3_C_2_T_x_ (Gly-MXene/Cys-MXene) on H-plasma-treated ITO	MXene PCE 24.96% Gly-MXene PCE 24.53% Cys-MXene PCE 24.92% *V_oc_* 726.6 ~ 731.7 mV *J_sc_* 39.96 ~ 40.31 mA/cm^2^ FF 82.19 ~ 85.66% WF tuning range 4.53~4.03 eV	Spray-coated double-sided MXene electrodes in SHJ solar cells as a silver paste replacement, with the WF lowered to 4.03 eV by electron-donating amino acid surface modification. Enhanced oxidation resistance, retaining over 90% of the initial efficiency after 200 days unencapsulated (25 °C, 60% RH).	[141]
	BME–Ti_3_C_2_T_x_ flake/MoS_2_ FET	HCl/LiF wet etching The colloid was mixed and sonicated with BME aqueous solution, allowing –SH groups to adsorb onto unsaturated Ti atoms at MXene edges and surface defects	β-Mercaptoethanol (BME)	WF 4.70 → 4.39 eV Conductivity (pristine) 3534.1 → 4514.9 S/cm (BME 0.05 M)	Ti–S bonds from the –SH group of BME suppress oxidation and lower the WF from 4.70 to 4.39 eV, aligning with the MoS_2_ CB. Ohmic contact with 10^6^ on/off ratio, 10 times ON current, and long-term stability.	[142]
	ITO/PAH·MXene/Al-doped ZnO	LiF/HCl etching N-doping with amine groups *via* reaction with a PAH aqueous solution	PAH-functionalized Ti_3_C_2_T_x_ MXene	WF 4.08~5.33 eV MXene *E_D_* 0.05 → −0.24 eV (PAH-functionalized)	Electron-donating amine groups from PAH lower the MXene WF by 0.24 eV, making the MXene/ZnO junction nearly Ohmic. Unfunctionalized junctions lose RR with Al doping; PAH-functionalized ones remain nonrectifying.	[137]
	InGaN/patterned Al_2_O_3_ insulating layer/two lateral Ti-Au Ohmic electrodes + Ti_3_C_2_T_x_ MXene Schottky contact (coated on the exposed InGaN region)—lateral MSM-type photodetector	LiF/HCl etching ascorbic acid added to a 0.3 mg/mL MXene solution, sonicated, and heat-treated at 65 °C for 2 h to reduce surface −OH groups	Ti_3_C_2_T_x_-C_6_H_8_O_6_	WF 4.20 → 4.34 eV heterojunction *Φ_B_* 0.56 → 0.70 eV *qV*_0_ 0.16 → 0.30 eV Responsivity 0.133 A/W (at −1 V, 420 nm) D* 2.81 × 10^11^ Jones (at −1 V)	Ascorbic acid treatment removes −OH groups, raising the WF from 4.20 to 4.34 eV and the SBH from 0.56 to 0.70 eV for a stronger built-in field and better carrier separation. ~5× higher responsivity (0.133 A/W) and faster response (37.49/110 µs) than the untreated device, with over 95% performance retained after 3 months.	[92]
	Normal OSC: ITO/U-MXene (HTL)/PBDB-T:ITIC/Ca/Al Inverted OSC: ITO/UH-MXene (ETL)/PBDB-T:ITIC/MoO_3_/Al	LiF/HCl wet etching UVO treatment oxidizes C element to increase work function N_2_H_4_ vapor treatment reduces C element with N-doping to decrease work function	U-MXene (UVO-treated, HTL), UH-MXene (UVO then N_2_H_4_-treated, ETL)	WF tuning range 4.08–4.95 eV Normal OSC: PCE 9.02% *J_sc_* 15.98 mA/cm^2^ *V_oc_* 0.89 V/FF 0.64 Inverted OSC: PCE 9.06% *J_sc_* 17.36 mA/cm^2^ *V_oc_* 0.87 V/FF 0.60	Work function of Ti_3_C_2_T_x_ was tuned from 4.08 to 4.95 eV by oxidizing/reducing the C element *via* UVO/N_2_H_4_ treatment, enabling use as HTL and ETL in non-fullerene OSCs. U-MXene as HTL and UH-MXene as ETL achieved PCEs of 9.02% and 9.06%, respectively, comparable to conventional buffer materials.	[93]
	Ag/SnCl_4_-treated MXene/Pyramid-textured *n*-Si/In:Ga	LiF/HCl etching Drop-casting MXene on *n*-Si and air drying → Spin-coating SnCl_4_ solution	SnCl_4_-treated MXene	PCE 4.7 (untreated pyramid) → 9.3% (optimal SnCl_4_-2 treatment) *J_sc_* 26.8 → 29.1 mA/cm^2^ *V_oc_* 423 → 585 mV FF 41.3 → 54.8% *R_s_* 1240 → 190 Ω/sq *J*_0_ 1.55 → 0.02 mA/cm^2^	SnCl_4_ treatment *via* Sn^4+^ interaction with MXene surface groups lowers the sheet resistance from 1240 to 190 Ω/sq and reduces Si/MXene interfacial defects, doubling the PCE from 4.7% to 9.3% in pyramid-textured devices. 88% of the initial PCE retained after 30 days in air, versus 67% untreated.	[143]
Nb_2_CT_x_	ITO/SnO_2_/O–Nb_2_C/Perovskite/Spiro-OMeTAD/Ag	HF etching Thermal oxidation (-F/-OH → -O)	(-O) terminations of the oxidized MXene derivative (O-Nb_2_C) and Nb_2_O_5_ nanoparticles	*V_oc_* 1.167 V *J_sc_* 24.86 mA/cm^2^ FF 0.80 PCE 23.20% HOMO −8.54 eV LUMO −4.34 eV	O-Nb_2_C at the SnO_2_ interface, optimizing energy levels and promoting uniform perovskite crystallization. Suppressed interfacial defects, giving 23.20% efficiency and 1500 h long-term stability.	[102]

**Table 5:** MXene-based transparent conducting electrodes.

Materials	Applications	Device Structure	Performance	Characteristics	Ref.
Ti_3_C_2_T_x_	Touch panels, healthcare sensors and wearable applications	MXene-based TCEs (30 mm × 30 mm)/thin PET film layer/MXene-based TCEs (30 mm × 30 mm)	Transparency: 60% (550 nm wavelength) Sheet resistance: 200 Ω/sq	The sheet resistance increases by only 0.16% at a 5 mm bending radius and by 5% after 1000 bending cycles at the same radius.	[147]
	Flexible and transparent display devices	PET/MXene/PDLC/MXene/PET	Sheet resistance: ~550 Ω/sq (TCE) Transmittance: ~80% (at 550 nm) (TCE), ~75% (PDLC-based smart windows)	At a bending radius of ~7.5 mm with a strain of 0.8%, the resistance change (Δ*R*/*R*_0_) remained low at approximately 0.7%. Additionally, at a bending radius of ~9.5 mm, variations in ΔT, T_ON_, T_OFF_, and C_r_ remained below 5% even after 10,000 bending cycles.	[148]
	Transparent Joule heaters and supercapacitors	Glass (or PET)/Ti_3_C_2_T_x_ film	Transmittance: 83.4% DC conductivity: 19,325 S/cm	Using blade-coated films instead of spin-coated ones results in a more compact and highly aligned flake structure, leading to improved DC conductivity and overall optoelectronic performance.	[5]
	Electrochromic devices	PET/MXene/PProDOT/electrolyte/MXene/PET	Transmittance: ~92.5% Sheet resistance: 53.8 Ω/sq FoM_e_: ~84 Cycling stability: >38,000 cycles	By confining MXene flakes within nanoimprinted grid channels, the electrode achieves high transmittance with low sheet resistance while maintaining excellent mechanical stability during repeated bending and twisting.	[149]
	Smart windows	PET/MXene/PDLC/MXene/PET	Transmittance: ~87% Sheet resistance: ~424 Ω/sq Device: Flexible smart window (high opacity in the OFF state/high transparency in the ON state) Threshold voltage: <10 V	The MXene-coated PET film shows a resistance change of ~30% over 28 days under ambient conditions, while exhibiting only ~1.5% relative resistance change during the 1000th bending cycle and a cumulative resistance change of ~20% after 1000 cycles at a ~3.9 mm radius.	[48]
	Soft robots and flexible wearable devices	LDPE/PI/H-MXene/Ag NWs	Photothermal: +40 °C (1.0 sun illumination) Electrothermal: +70 °C (1 V in the dark) Crawling speed: 0.042 mm/s (soft robot) Power density: 3.3 W/cm^2^ (soft robot)	MXene-based coating that exhibited high transparency in visible light and, furthermore, introduced hierarchical wrinkled microstructures through chemical treatment to reduce the surface reflectivity and increase the conductivity. Presents a novel strategy for the development of a heat generation device achieving high-performance photothermal and electrothermal conversion.	[146]
	Transparent device applications	MXene@BZT/UHMWPE	Transmittance: over 85% Haze: <12% Photothermal: 65 °C under 400 mW/cm^2^ light irradiation	The film provides annual cooling energy savings of 31–61 MJ/m^2^ (3–12% of total refrigeration energy) and lowers indoor temperature by 6–7 °C in a covered glass model, demonstrating its potential for transparent energy-saving applications.	[150]

**Table 6:** MXene-based solar cells.

Materials	Applications	Device Structure	Performance	Characteristics	Ref.
Ti_3_C_2_T_x_	PSCs IPSCs OSCs	Glass-ITO/c-TiO_2_/MXene@TiO_2_/perovskite/Spiro-MeOTAD/Au	PCE: 19.3% (Control: 17.46%) J_sc_: 23.79 mA/cm^2^ (Control: 22.88 mA/cm^2^) V_oc_: 1.08 V (Control: 1.06 V) FF: 0.754 (Control: 0.72)	The multifunctional MXene@TiO_2_ ETL enhances PSC efficiency and stability through increased TiO_2_ conductivity and improved surface potential and crystallization of the perovskite layer.	[151]
		FTO/c-TiO_2_/m-TiO_2_/HP:H3pp/MXene:H3pp/Spiro-OMeTAD/Au	PCE: ≈22% (Control: 20.57%) J_sc_: 24.68 mA/cm^2^ (Control: 24.14 mA/cm^2^) V_oc_: 1.142 V (Control: 1.108 V) FF: 77.9% (Control: 76.9%)	Addition of the H3pp organic additive to MXene and HP exhibits combined effects in ion migration inhibition and defect passivation. MXene-based interface engineering facilitates device recovery under outdoor stress conditions, while this work reports the first ISOS-O outdoor stability assessment of MXene-based PSCs.	[152]
		FTO/SnO_2_/MXene/Cs_0.05_(FA_0.83_MA_0.17_)_0.95_Pb(I_0.83_Br_0.17_)_3_/Spiro-OMeTAD/Ag/ITO	PCE: 14.78% AVT: 22.68% (AVT_800_: 26.70%)	MXene was inserted between the SnO_2_ ETL and the perovskite layer rather than being incorporated into the SnO_2_ ETL. Long-term ambient stability: retained 85.58% of initial PCE after over 1000 h (unencapsulated).	[153]
		ITO/SnO_2_/MXene:m-SWCNTs/Perovskite/Spiro-OMeTAD/Au	PCE: 21.42% (Control: 18.84%) J_sc_: 25.09 mA/cm^2^ (Control: 24.71 mA/cm^2^) V_oc_: 1.073 V (Control: 1.043 V) FF: 0.80 (Control: 0.73)	Passivating oxygen-vacancy-related defects in SnO_2_ with the MXene/m-SWCNTs interfacial layer. The highest-performing device was achieved by introducing a hybrid interfacial layer combining MXene and m-SWCNTs at a 2:1 (w/w) ratio to compensate for MXene conductivity loss due to oxidation.	[154]
		FTO/c-TiO_2_/m-TiO_2_-TQD/TQD-perovskite/Spiro-OMeTAD:Cu_1.8_S/Au	PCE: 21.64% (Optimized with TQD and Cu_1.8_S)	Introducing TQD into the TiO_2_ ETL and perovskite layer reduces interfacial defects and enhances perovskite crystal growth and conductivity, thereby suppressing recombination and improving carrier separation and transport.	[155]
		Glass/FTO/MXene/SnO_2_/Perovskite/Spiro-OMeTAD/Au	PCE: 20.65%	MXene–SnO_2_ interaction and electron hybridization enhance electron mobility and enable favorable band alignment for efficient charge transfer. Hydrophobic and smooth FTO/MXene/SnO_2_ surface supports the growth of low trap density perovskite films.	[156]
		Glass/ITO/ZnO (or ZnO/Ti_3_C_2_T_x_)/BHJ active layer/MoO_3_/Ag	PBDB-T:ITIC PCE: average 12.20% (Control: 10.56%) J_sc_: average 18.63 mA/cm^2^ V_oc_: 0.93 V FF: 70.39% PM6:Y6 PCE: average 16.51% (Control: 14.99%) J_sc_: average 26.38 mA/cm^2^ V_oc_: 0.83 V FF: 75.40%	The optimal amount of Ti_3_C_2_T_x_ is 0.05 wt%. The transmittance in the UV region decreases as the amount of Ti_3_C_2_T_x_ increases from 0.01 to 0.07 wt%. Ti_3_C_2_T_x_ enhances ZnO conductivity and charge transfer by creating additional transport pathways. Zn–O–Ti bond formation reduces surface defects, suppresses charge recombination, and may prolong exciton lifetimes.	[157]
Nb_2_CT_x_		ITO/PEDOT:PSS-Nb_2_C/active layer/BCP/Al	PCE: 19.33%	Enhanced long-term stability: PEDOT:PSS-Nb_2_C device maintained 82% of initial PCE after 350 h (Control: 72%) under N_2_ atmosphere.	[158]

**Table 7:** MXene-based photodetectors.

Materials	Applications	Device Structure	Performance	Characteristics	Ref.
Ti_3_C_2_T_x_	Photodetectors	Thermochromic composite/PI/Au/MXene	Responsivity: 0.33 mA/W	Dual-mode infrared imaging advances human–machine collaborative optoelectronics, while MXene-based photothermal conversion enables thermochromic color changes for visual detection.	[159]
		GaN/MXene thin film and MXene thick film	Responsivity: 81 mA/W Response speed: less than 31 and 29 ms Photocurrent: 397 nA (optical power density 0.039 mW/cm^2^), 60 μA (optical power density 38.19 mW/cm^2^) Ultra-high on/off ratio: 1.33 × 10^6^	This work proposes a general strategy for MXene/semiconductor Schottky junction-based self-powered photodetectors, where transmittance contrast-induced asymmetric carrier generation and injection produce a photocurrent at zero bias.	[160]
		Glass/ITO/ZnO+MXene/PbS QD-inks/PbS-EDT/Au	Ultra-high light-to-dark current ratio: ~10^5^ Dark current density: 8.33 × 10^−4^ mA/cm^2^ Specific detectivity (D*): 1.99 × 10^11^ Jones at 0 V Responsivity: 0.12 A/W	Interface-engineered QD-based SWIR photodetectors enhance optoelectronic performance, while 2D MXene improves device performance by passivating ZnO defects, suppressing recombination, and promoting interfacial carrier transport.	[161]
		GaN/(Ti_3_C_2_T_x_/Au and Ti/Al/Ni/Au)	Responsivity: 681.6 mA/W Specific detectivity: 7.65 × 10^13^ Jones (zero bias) Ultraviolet/visible rejection ratio: 3.9 × 10^3^ SNR: 2.4 × 10^5^ Rise time: 0.43 ms Decay time: 0.68 ms	The fast response enables the device to function as a UV receiver that detects pulsed signals in an optical sensing system.	[162]
		Alumina base/Au/Bi_2_S_3_-Ti_3_C_2_T_x_ composite	Responsivity: 36.7 A/W (300 nm), 26.2 A/W (780 nm) EQE: 3.9 × 10^3^% Response time: 300 μs (3 V bias)	This work elucidates enhanced charge transport and ultra-broadband photodetection in MXene-based composites for high-performance optoelectronics.	[163]
		TC/CNC	Max Temperature: 182.3 °C (at 0.45 W/cm^2^) Heating Time: ≤1 s Surface Roughness (Ra): 41.2 nm (Pure TC: 15.9 nm)	A hydrophilic, nacre-like aligned free-standing film fabricated by vacuum filtration enhances photothermal conversion through increased surface roughness, reducing reflection and extending light paths, enabling rapid heating under low NIR irradiation.	[164]
Nb_2_CT_x_		Nb_2_C-ITO/Platinum wire/Ag/AgCl	Responsivity: 12.6 μA/W (365 nm) Response/Recovery time: <0.15 s Narrow-band response: 350–400 nm Photocurrent density: 850 nA/cm^2^ Photocurrent decrease: 0.015% per cycle over 1000 cycles	Few-layer Nb_2_C MXene exhibits a wavelength-dependent narrow-band UV photoresponse, while its photodetection performance can be effectively tuned by the applied bias potential and electrolyte concentration, demonstrating strong potential for narrow-band photodetectors.	[165]
		Glass/Nb_2_CT_x_/MAPbI_3_/Au	Responsivity: 0.25 A/W Temporal photoresponse: <4.5 μs On/Off ratio: ~10^3^ (NIR laser 1064 nm) Response times: <30 ms (NIR laser 1064 nm)	This work sheds light on the potential of the perovskite/MXene platform and stimulates the need for further research on optoelectronic applications of MXenes.	[166]

**Table 8:** MXene-based light-emitting devices.

Materials	Applications	Device Structure	Performance	Characteristics	Ref.
Ti_3_C_2_T_x_	OLEDs QLEDs PLEDs PeLEDs	FTCE/MoO_3_/TAPC/EML/TPBi/LiF/Al	EQE: 22.9% (green), 24.6% (red) Current efficiency: 81.4 cd/A (green), 28.4 cd/A (red)	FTCE: P-MXene/AgNWs/MXene. Sheet resistance remains nearly unchanged after annealing at 160 °C for 3.5 h and after 1000 bending cycles. At luminance of 2027 cd/m^2^, CE and EQE remain at 76.3 cd/A and 21.5%, respectively, with an efficiency roll-off of only 6.7%.	[167]
		MXene/PEDOT:PSS/Organic layers/Al electrode/Al_2_O_3_/TiO_2_ nanolaminate/S−H nanocomposite	Luminance: 1691 cd/m^2^ (red), 1377 cd/m^2^ (green), 1475 cd/m^2^ (blue) Sheet resistance: 82.3 Ω/sq Current density: 40.4 mA/cm^2^ (red), 4.26 mA/cm^2^ (green), 18.6 mA/cm^2^ (blue)	Stable operation without dark spots was maintained after storage in air for over 2000 h, 6 h of water immersion, and folding at strains up to 1.7%.	[168]
		Glass substrate/m-MXene/PEDOT:PSS/Super Yellow/LiF/Al	High WF: 5.84 eV Sheet resistance: 97.4 Ω/sq	Removing contaminants and –OH surface terminations enhances conductivity and WF, while PFSA further improves charge injection and WF. The developed MXene electrode showed nearly unchanged R_S_ and maintained a WF above 5.60 eV after 22 days of air exposure.	[169]
		MXene/PVPh/PEDOT:PSS/PDY-132/MWNTs/LiF/Al	Luminance: ~7500 cd/m^2^ (after 7 d of air exposure) Maximum luminance: ~9500 cd/m^2^	PVPh passivation suppresses MXene oxidation, enabling reliable organic AC-EL operation even after 7 d of air exposure. The flexible organic AC-EL display maintained stable operation over 1000 bending cycles at a bending radius of 7.5 mm.	[170]
		AgNW-MP21 FTEs/F4-TCNQ/2-TNATA/NPB/(2,3-dpqx-F_2_)_2_Ir(pzrl)/Alq_3_/LiF/Al	Sheet resistance: 17 Ω/sq Transmittance: 97.6% (550 nm) EQE: 25.9% Luminance: 2303 cd/m^2^ Luminous efficiency > 21 cd/A	The MP21 layer raises the AgNW FTE work function by 0.2 eV, lowering the interfacial barrier for improved hole injection, while enhancing electrode performance by increasing conductivity and reducing surface roughness.	[171]
		PET/MXAg@PMMA/ZnO/PVK/CdSe@ZnS/ZnS/PEIE/poly-TPD/MoO_x_/Al	Sheet resistance: 13.9 Ω/sq Transmittance: 83.8% Current efficiency: 25.8 cd/A EQE: 9.88% Luminance: 31,340 cd/m^2^	Exceptional bending stability with only 0.8% luminance variation after 1000 cycles, retaining 99% of the initial luminance (3575 cd/m^2^). Outperformed ITO-based QLEDs (~30% loss) and AgNW-based QLEDs (~7% loss).	[172]
		ITO/PEDOT:PSS/TFB/QDs/ZnO/MXene/Ag NWs	Transmittance: 84.6% (620 nm), 86.4% (average visible light) Sheet resistance: 16.07 Ω/sq Maximum CE: 23.12 cd/A Maximum EQE: 13.98% Maximum brightness: 21,015 cd/m^2^	It improves contact welding, lowers interfacial resistance between Ag NWs, and enhances charge transfer at the ZnO–Ag NW interface.	[173]
		MXene/PEDOT:PSS/PDY-132/LiF/Al	Turn-on voltage: 2.1 V Current efficiency: 7 cd/A Brightness: 12,547 cd/m^2^	The device maintains a luminance of ≈200 cd/m^2^ at 6 V and 1 kHz when bent at a 7.5 mm radius, and retains ≈90% of its initial brightness after 1000 bending cycles at the same radius.	[174]
		Glass/ITO/ZTC/PEI/NCs/TCTA/MoO_3_/Au	EQE: 17.4% (using ZTC containing 10% Ti_3_C_2_) V_ON_: 2.28 V	The EQE_max_ increases from 14.2% (0%) to 15.3% (5%) and 17.3% (10%), then decreases to 11.5% (20%), while the half-lifetime improves from 9 min (0%) to 23 min (10% Ti_3_C_2_).	[175]
	Wearable Optoelectronics	PET/ITO/MXene/BP/ITO/PET	Sensitivity = 77.61/kPa	Dual-functionality *via* nano-intercalation: periodic insertion of BP between MXene layers expands *d*-spacing to facilitate ion diffusion while creating a low-modulus structure for high deformability.	[176]
		PET/Ti_3_C_2_ MXene/n-GraHIL/TAPC/TCTA:Ir(ppy)_2_acac and CBP:Ir(ppy)_2_acac/TPBi/LiF/Al	V_on_ = 3 V EQE = 28.5%	The hole-injection barrier was physically lowered by removing work function-degrading -OH groups through thermal treatment, while charge flow was optimized by adopting a neutral hole-injection layer to protect the acid-vulnerable MXene electrode.	[177]
		PET/Cr-Au electrodes/CTS-MXene film/PDMS	V_on_ = 1.0 V I_max_ = 30 μA	Achieving high-sensitivity pressure and humidity sensing *via* chitosan-bridged MXene structure with low-voltage operation and excellent biostability.	[178]
		PET/Composite Electrode (0D AgNPs + 1D AgNWs + 2D MXene + 3D PEDOT:PSS)/PEDOT:PSS/Perovskite/TPBi/LiF/Al	Luminance = 45,166 cd/m^2^ EQE = 16.5%	Utilizing mixed-dimensional (0D–1D–2D–3D) components for high conductivity and efficient heat sinking.	[4]
		Paper substrate/Cu tape electrodes/MXene-BC film/PP film	V_on_ = 0.5 V I_max_ = 30 nA	Piezoresistive characteristics based on contact resistance variation within a 3D network according to external pressure and vibration.	[179]
		PEN/ITO/MG-PEDOT/Perovskite/PCBM/BCP/Ag	V_oc_ = 1.18 V PCE = 20.25%	Self-assembled vertical-gradient structure: MXene is concentrated at the top of the hole transport layer (HTL) to align energy levels, while the underlying glucose caramel layer assists in perovskite crystal growth.	[180]
		LIG/leather/MXene-LP/PU	V_oc_ = 199.56 V I_charge_ = 6.78 μA	Natural leather-mimetic structure: Enhancing mechanical strength through hydrogen bonding between MXene and the collagen fibers of leather, and implementing self-powered sensing by utilizing the principle of triboelectric nanogenerators (TENG).	[181]
		Au NCs/MXene/GO Fiber	P = 0.1 mW LOD = 10^−11^ Mf	Plasmonic coupling: maximizing the signal through the synergetic effect of electromagnetic enhancement (EM) between Au nanoclusters and chemical enhancement (CM) *via* charge transfer from MXene.	[182]
		PDMS/Cu electrode/MXene@P(VDF-TrFE)/Cu electrode/PDMS	V = 1 V I = 0.06 mA	Piezoresistive effect: resistance variation according to changes in interlayer distance and contact points.	[183]
		Medical adhesive tape/MXene/cotton fiber/PET-Cu interdigitated electrode/Medical adhesive tape	V = 1 V I = 10^−4^ A	Piezoresistive effect: utilizing the wide interface contact area between fibers.	[184]
Nb_2_CT_x_		PET/ITO/Nb_2_CT_x_@Bi/KOH/Pt	V_on_ = 0 V V_bias_ = 0.8 V	Self-powered photodetection *via* Schottky heterojunction: utilizing the built-in electric field between high-work function MXene and low-work function Bismuth to separate photo-induced charges without external bias.	[185]
V_4_C_3,_ V_2_C		Nylon Membrane/V_4_C_3_, V_2_C MXene layer/analyte molecules	LOD = 5 nM	2D downsizing and molecular enrichment: Enhancing the density of states (DOS) by thinning MXene and maximizing sensitivity by ultra-rapidly enriching analyte molecules on the surface *via* vacuum filtration.	[186]

**Table 9:** Self-powered MXene optical sensors and energy harvesting.

Materials	MXene’s Role	Device Structure	Mechanism	Performance and Stability	Application	Characteristics	Ref.
Ti_3_C_2_T_x_	Conductive electrode/dielectric filler	PDMS-MXene/Si/SiO_2_/Au-MoS_2_-Au	Triboelectric nanogenerator (TENG)	V_oc_ = 14 V I_sc_ = 7 μA Flexible wristband, validated over 100 trials	Heart rate monitoring (PPG)/UV detection	First report of TENG-coupled photodetector for real-time wearable heart rate monitoring Single wristband integrating energy harvesting, heart rate, and UV sensing 1 wt% MXene reduced impedance from 100 MΩ to 10 MΩ, matching MoS2 photodetector	[197]
	Nucleating agent/polarization enhancer	PET/ITO/MXene/MAPbI_3/_Au	Piezoelectric nanogenerator (PENG)	V = 3.71 V J = 0.203 μA/cm^2^ *d*_33_ = 24.15 pm/V Long-term stability	Self-powered photodetector/wearable sensor	First report of MXene enhancing intrinsic piezoelectricity of MAPbI_3_ *via* MA^+^ dipole alignment, confirmed by DFT −1.2% compressive strain caused 102% photocurrent increase with millisecond response time	[198]
	*ß*-phase inducer/charge trapping site	PET/Al/PVDF-AlN@MXene-CFO/Al/PET	Magneto-mechano-electric (MME) hybrid	V_oc_ = 18.1 V I_sc_ = 1.24 μA P_max_ = 2.3 μW/cm^2^ 3000-cycle stability/flexibility	Wireless power transfer (6cm)/IoT sensor node	First report of Ti_3_C_2_T_x_-CFO hybrid integrated into piezoelectric polymer-ceramic nanofiber for multiferroic composites MXene surface groups promoted PVDF *β*-phase to 84.20% and d_33_ to 32.67 pC/N *via* dipole alignment Dual-mode harvesting of magnetic noise and mechanical energy validated by COMSOL simulation	[199]
	Conductive framework/charge storage reservoir	Cu/PET/MXene-GO-Siloxene/Textile	Triboelectric nanogenerator (TENG)	V_oc_ = 380 V I_sc_ = 63 μA P_max_ = 627 μW/m^2^ 12,000-cycle stability	Smart clothing/self-powered motion sensor	Novel citric acid covalent bonding strategy of MXene onto textile resolved nanofiller agglomeration Same MXene material group used for both TENG and MSC, reducing system complexity Integrated device self-charged to 2.4 V in 110 s with 95 ± 1% retention after 12,000 cycles	[200]
	Ion transport channel/hygroscopic enhancer	PFA/Cu/CNF-PVA-MXene aerogel/Cu/organo-ionic hydrogel/Al	Moisture-induced and triboelectric hybrid	V_moisture_ = 0.6 V V_TENG_ = 106 V I_sc_ = 90 μA 1000 cycles at 90% RH	All-weather energy harvester/humidity sensor	First resolution of moisture conflict between MEG and TENG using wood-inspired biomimetic MXene aerogel MXene nanochannel enabled selective cation transport and microcapacitor effect boosted TENG output Eco-friendly recyclable device achieving 77 μW/cm^2^ across 20–90% humidity	[201]
	Photothermal agent/electrically conductive filler	PAN-MXene fiber/yarn	Photothermal-Thermoelectric Hybrid	P_max_ = 432.7 mW/m^2^ 12,000-cycle TENG stability	Thermal management/self-powered temp sensor	MXene as multifunctional filler integrates thermal, photothermal, triboelectric, and tactile sensing in single electrospun yarn MXene lowered PAN surface potential to −360 mV, enhancing tribo-negative character	[202]
Ti_3_C_2_T_x_	Charge trapping layer/conductive filler	PET/conductive sponge/MXene-silicone nanocomposite	Noncontact triboelectric sensing	V_oc_ = 251.8 V Q_sc_ = 65.3 nC Stable output for 5000 s continuous approach/separation	Intelligent noncontact switch/human motion monitoring	3 wt% MXene/silicone + embedded conductive sponge achieves record 2m noncontact sensing distance MXene nanosheets enhance charge trapping, increasing V_oc_/Q_sc_ by ~90% First application as self-powered noncontact sensor for vehicle sentry mode and blind-spot detection	[203]
	Bifunctional conductive filler/polarization inducer	Al/PI/MXene-BaTiO_3_-PDMS	Piezo-triboelectric hybrid	V_oc_ = 80 V P_max_ = 13.5 W/m^2^ 20,000-cycle stability	Portable electronics power source	MXene as bifunctional filler in BaTiO_3_/PDMS achieves 80 V/13.5 W/m^2^ MXene forms continuous electric flux path, boosting V_oc_ 200% after poling Self-powered HMI for robot hand control and material detection with 93.33% accuracy	[204]
	Active piezoelectric layer	Au/oxidized MXene/Au	Piezoelectric and Piezotronics	P_max_ = 31.04 mW/m^2^ Conversion efficiency = 17.4% 1000-cycle stability	Nanoscale sensors/Piezotronic devices	Inversion symmetry breaking at Ti_3_C_2_T_x_-O stage boosts piezoelectric output ~3× to 1.15 nA Achieves 31.04 mW/m^2^ and 17.4% efficiency, surpassing all previously reported 2D materials Oxidation-controlled piezoelectric tuning demonstrated for stretchable nanodevice applications	[205]
	Conductive filler/ferroelectric coupling enhancer	AgNW/TPU-BTO-MXene/PDMS/AgNW	Ferroelectricity-coupled triboelectric	V_oc_ = 260 V P_max_ = 6.5 W/m^3^ Stretchable to 60% strain	Self-powered stretchable electronics	BTO-MXene ferroelectric coupling achieves high *ε*r~30 with low tan *δ*~0.24 Hierarchical porous structure achieves 260 V/6.65 W/m^2^/79% energy conversion efficiency Stretchable self-powered sensor enables pulse monitoring, acoustic sensing, and robotic gesture recognition	[206]
	Conductive network/surface roughness enhancer	Conductive fabric/NPCO-silicone/MXene-silicone/conductive fabric	Triboelectric nanogenerator (TENG)	V_oc_ = 1680 V P_max_ = 10.4 W/m^3^ I_sc_ = 117 μA Washable and durable; stretchability	Wearable self-powered sensing	MXene/silicone bifunctional interlayer boosts surface potential 9× vs. pure silicone NPCO/silicone + MXene double-layer achieves 10.4 W/m^2^, 23× higher than pure silicone 230% stretchable wearable TENG with 5.82 V/kPa sensitivity for foot pressure mapping and wearable keyboard	[207]
	Growth substrate/conductive electrode	ITO-PET/Li:ZnO NW- Mxene composite/ITO-PET	Piezoelectric nanogenerator (PENG)	V_oc_ = 10 V P_max_ = 9 W/m^3,^ Flexible and stable output	Self-powered pressure sensor	Li:ZnO NWs on Ti_3_C_2_ form semiconductor-metal hybrid, enhancing dielectric constant and poling efficiency Ti_3_C_2_ local ground effect achieves *d*_33_ eff 22.1 pm/V and ~9 μW/cm^2^ power density, 2× improvement Flexible self-powered PENG demonstrated as wearable motion sensor for finger, walking, and impact detection	[208]

**Table 10:** MXene-based artificial synapses for optical memory and neuromorphic vision.

Materials	Device Structure	Performance	Mechanism	Characteristics	Application	Ref.
Ti_3_C_2_T_x_	Au/Ti_3_C_2_T_x_/Y:HfO_2_(40 nm)/FTO/Glass	R_off_/R_on_: >10^3^ Analog retention: >10^4^ s CNN MNIST: 97.1% (optoelec.)	CF rupture + interface potential barrier height + ferroelectric polarization	Digital + analog dual switching in one device Optoelectronic co-stimulation improves nonlinearity from 5.83 → 1.03 Single device realizes AND/OR logic gates and 3-person humidity-veto voting logic	Logic-in-Memory Optoelectronic Synapse Multi-sensor Integration	[213]
	Au/LPE/Ti_3_C_2_-MXene/Si	Power (min.): 460 fW Min. spike sensitivity: ±10 mV PSC range: 1.2–3.2 nA	Li^+^ ion migration and adsorption/insertion	World-record power consumption of 460 fW *via* Li^+^ intercalation into MXene interlayers ±80 mV ultra-sensitivity Bidirectional plasticity	Artificial Peripheral Nervous System Dendritic Integration	[214]
	Al(S/D)/ZTO/TiO_2_/MXene/SiO_2_/Al(gate)	NL_LTP/LTD_ (UV + elec.): 0.51/1.73 NL_LTP/LTD_ (elec. only): 0.73/2.05 Cyclic stability: 121 cycles (48,400 pulses)	Electron trapping/de-trapping *via* TiO_2_ tunneling layer	Self-oxidized TiO_2_ on MXene serves as both floating gate and tunneling layer First UV-responsive MXene FGT with Pavlov conditioning and supervised learning	Bio-imitation Vision Supervised Learning	[215]
	Cu/Ti_3_C_2_/PZT(40 nm)/Pt/Si	R_off_/R_on_: 10^6^% SET voltage: 1.5 V Retention: >3000 s MNIST: 95.13%	Cu CF + ferroelectric polarization barrier	MXene reduces Cu diffusion barrier from 4.43 → 0.88 eV (DFT confirmed), enabling SET voltage of 1.5 V LTP/LTD/STDP/PPF all realized without extra circuitry	Neuromorphic Computing Pattern Recognition	[216]
	Al(S/D)/ZTO\GMX(fd)-ZTO/SiO_2_/Si(gate)	EPSC: ~230 nA (blue, multi-spike) Conductance States: 256 levels per RGB channel MNIST: 98.3% (blue)/98.1% (green)/96.8% (red) G_max_: 304.4 nS	GeO_x_ V_o_ activation + GMX/ZTO heterostructure carrier trapping	GeO_x_-coated MXene overcomes visible-light limitation of bare MXene 256-level RGB color mapping NCP neural network for autonomous driving	In-memory Computing Visual Perception Autonomous Driving	[217]
	PI/m-CNTs(gate)/HPI(40 nm)/MXene(10 nm)/Al_2_O_3_(7 nm)/s-CNTs/m-CNTs(S/D)	V_op_ (min): 0.2 V On/off ratio: 1.52 × 10^3^ Hysteresis window: 4.9 V SS: 0.42 V/dec Bending: r = 4 mm 1000 cycles stable MNIST: 92.0%	Electron tunneling: s-CNT ↔ MXene FG *via* Al_2_O_3_	First flexible MXene FGT 0.2 V lowest op. voltage in flexible FGTs Stress-dependent Pavlov conditioning simulated under bending	Wearable Neuromorphic Cognitive Learning	[218]
	Au/Chiral PVK NW [(R/S-MBA)_2_PbI_4_]/Ti_3_C_2_T_x_/SiO_2_(300 nm)/Si	R_max_: 2.3 A/W Carrier lifetime: 36 ps → 68 ps (w/MXene) RC NRMSE: 0.023 (Mackey–Glass)	Chiral CD/LD + MXene hole extraction → prolonged carrier lifetime (36→68 ps)	MXene hole transport extends carrier lifetime from 36 → 68 ps, enabling synaptic behavior First full-Stokes polarization-resolved PAS with CD > 400 mdeg Reservoir computing NRMSE = 0.023	Full-Stokes Polarimetry Reservoir Computing Neuromorphic Vision	[219]

## Data Availability

No new data were created or analyzed in this study.
